# Untargeted Metabolomics Identifies Key Metabolic Pathways Altered by Thymoquinone in Leukemic Cancer Cells

**DOI:** 10.3390/nu12061792

**Published:** 2020-06-17

**Authors:** Asma Ahmed AlGhamdi, Mohammed Razeeth Shait Mohammed, Mazin A. Zamzami, Abdulrahman L. Al-Malki, Mohamad Hasan Qari, Mohammad Imran Khan, Hani Choudhry

**Affiliations:** 1 Biochemistry Department, Faculty of Science, King Abdulaziz University, Jeddah 21589, Saudi Arabia; prof.asmaahmed@gmail.com (A.A.A.); razeeth.new@gmail.com (M.R.S.M.); mzamzami@kau.edu.sa (M.A.Z.); alalmalki@kau.edu.sa (A.L.A.-M.); 2 Cancer Metabolism and Epigenetic Unit, Biochemistry Department, Faculty of Science, King Abdulaziz University, Jeddah 21589, Saudi Arabia; 3 Cancer and Mutagenesis Research Unit, King Fahd Medical Research Center, King Abdulaziz University, Jeddah 21589, Saudi Arabia; 4 Hematology Department, Faculty of Medicine, King Abdulaziz University Hospital, King Abdulaziz University, Jeddah 21589, Saudi Arabia; drqari200@gmail.com

**Keywords:** thymoquinone, leukemia, metabolites, LC-MS/MS, metabolism, DNA damage

## Abstract

Thymoquinone (TQ), a naturally occurring anticancer compound extracted from *Nigella sativa* oil, has been extensively reported to possess potent anti-cancer properties. Experimental studies showed the anti-proliferative, pro-apoptotic, and anti-metastatic effects of TQ on different cancer cells. One of the possible mechanisms underlying these effects includes alteration in key metabolic pathways that are critical for cancer cell survival. However, an extensive landscape of the metabolites altered by TQ in cancer cells remains elusive. Here, we performed an untargeted metabolomics study using leukemic cancer cell lines during treatment with TQ and found alteration in approximately 335 metabolites. Pathway analysis showed alteration in key metabolic pathways like TCA cycle, amino acid metabolism, sphingolipid metabolism and nucleotide metabolism, which are critical for leukemic cell survival and death. We found a dramatic increase in metabolites like thymine glycol in TQ-treated cancer cells, a metabolite known to induce DNA damage and apoptosis. Similarly, we observed a sharp decline in cellular guanine levels, important for leukemic cancer cell survival. Overall, we provided an extensive metabolic landscape of leukemic cancer cells and identified the key metabolites and pathways altered, which could be critical and responsible for the anti-proliferative function of TQ.

## 1. Introduction

In the last century, great advances were made in modern medicine to control diseases. However, many diseases, such as cancers, are not yet completely curable. To find out new and alternative therapies, researchers are working with traditional medicines (bioactive molecules), in parallel with modern medicine, to create new combinations for better treatment and management of diseases.

*Nigella sativa* (*N. sativa*), has been used for medicinal purposes for centuries [1]. *N. sativa* belongs to the botanical family of Ranunculaceae. It is a small shrub with tapering green leaves and rosaceous white and purplish flowers [2]. The most important bioactive elements found in *N. sativa* are; thymoquinone, thymohydroquinone, dithymoquinone, thymol, nigellimine-N-oxide, nigellicine, nigellidine, arvacrol, and alpha-hederin [2]. Among these, Thymoquinone (TQ) is an important bioactive ingredient primarily found in black seed oil. Recent scientific investigations on TQ indicate a number of bioactivities, which include anti-carcinogenetic, anti-inflammatory, antiulcer, antihypertensive, antibacterial and antifungal, hepatoprotective, antipyretic and analgesic, as well as antioxidant activities such as reducing reactive oxygen species, inhibition of rheumatoid arthritis in rat models, and antihyperlipidemic [3]. Treatment of cancer cells with TQ can result in inhibition of tumor cell proliferation within modulation of apoptosis signaling, inhibition of angiogenesis, and cell cycle arrest [4].

TQ has been shown to negatively modulate pyruvate kinase M2 (PKM2), an enzyme related to cancer cell energy pathways [5]. Similarly, TQ treatment has been shown to modulate various TCA cycle metabolites and lipids in cancer cells, which are critical for their survival. Further, TQ represses many signaling pathways directly involved in controlling the metabolic pathways of cancer cells, like PI3K, AKT, JNK and STAT3 [6].

System-wide analyses of metabolites under the umbrella of metabolomics allow a unique opportunity to understand the molecular aspects of carcinogenesis and cancer biology by enabling deep investigation of targeted aspects of cancer metabolism [7,8]. In addition, it provides a unique opportunity to understand and quantify a global impact of anti-carcinogenic compounds affecting the metabolism of cancer cells. The major aim of the current study is to explore the metabolic impacts of TQ treatment on cancer cells (leukemia cell lines), and to obtain the differences in their metabolomic patterns, in order to identify metabolites and modified metabolic pathways.

## 2. Materials and Methods

### 2.1. Cell Culture

Acute T cell leukemia (Jurkat (clone E6-1)), acute pro-myelocytic leukemia (HL-60), and an erythroleukemia cell line derived from a chronic myeloid leukemia patient (K-562) were obtained from the American Type Culture Collection (ATCC) (Rockville, MD, USA). These cells were grown as a suspension culture. These cells were cultured in Roswell Park Memorial Institute (RPMI 1640), supplemented with 15% heat-inactivated fetal bovine serum (FBS), and 1X penicillin–streptomycin. Cells were monitored daily using a microscope to monitor confluence and general culture conditions. Every two-days, the cells were passaged at a dilution of 1:1 or 1:2. Sub-culturing was done when the cell density was more than 1 × 10^6^ cells/mL. Frozen cell lines were stored in liquid nitrogen and thawed in a water bath for 30 to 60 s until the thawing was partially complete. Cell counting was done by using a hemocytometer.

### 2.2. TQ Preparation and Treatment

TQ solution was prepared in ethanol at a concentration of 100 µM. This stock was stored at −20 °C in eppendorf tubes wrapped in aluminum foil to avoid dimer formation. All cell lines were treated by TQ immediately after preparation and treated for 24 h using two different concentrations (5 µM and 10 µM) for metabolite extraction.

### 2.3. Measurement of Cell Viability Using Trypan Blue Exclusion Test

Trypan blue exclusion assay allows a direct identification and enumeration of live (unstained) and dead (blue) cells in a given population. however; it is not able to differentiate between apoptotic and necrotic cells. Jurkat, HL-60 and K-562 were plated in replicate (1.5 × 10^5^ cells/well) in a 96-well micro-plate and treated with TQ (5 µM and 10 µM), followed by an incubation of 24 h at 37 °C and 5% CO_2_. After incubation, 20 µL from cell and 20 µL from TB was taken and mixed, and the viable and non-viable cells were counted using a hemocytometer. The negative control cell suspension was treated with ethanol of different doses (5 µM and 10 µM).

### 2.4. Measurement of Growth Assay and Cell Proliferation Using WST-1 Assay

Briefly, 7 × 10^3^ cells/mL cells were seeded in a 96-well plate with a cell density of 1.5 × 10^5^ cells/mL, and were treated with TQ (5 µM and 10 µM). The WST-1 reagent was applied to all wells, with a final dilution of 1:10 for 4 h prior to examination of the cells after 24 h treatment. The WST-1 assay is based on the cleavage of water-soluble tetrazolium salt-1, which is tetrazolium salt that is reduced extra-cellularly to formazan dye by enzymes of the plasma membrane oxidoreductase. The primary reductant is NADH, derived from the TCA of the mitochondria. WST-1 is converted by metabolically active cells and was employed to measure cell proliferation. The optical density of the formazan dye was measured to get an estimate of live cells. The absorbance was measured at a wavelength of 450 nm, using the ELISA. The value obtained from the blank (complete medium only) was subtracted manually from all wells before calculating the percentage metabolism in each well.

### 2.5. Apoptosis Assay

Apoptosis was assessed by using the Annexin V-PI kit. Annexin V can specifically bind to phosphatidylserine. Cells undergoing apoptosis will experience diffusion of phosphatidylserine from inner cell membrane to outer cell membrane, whereby these proteins can be quantified by targeting them with fluorescence-tagged Annexin V. The experimental procedures were carried out according to the manufacturer’s protocol. Briefly, cells were seeded (2 × 10^6^ cells/well) in a 6-well microtiter plate followed by overnight incubation with and without treatment. After 24 h incubation, the cells were washed, and incubated with Annexin V-FITC solution for 15 min under dark conditions. After a washing step, the cells were analyzed with a flow cytometer after the adding of PI solution.

### 2.6. Cell Cycle Assay

Cell cycle progression of leukemia cells was examined by flow cytometry after propidium iodide (PI) staining. Cell cycle phases can be differentiated by measuring their DNA content, where G1 phase is 2n, S phase is between 2n and 4n, and G2 phase is 4n. PI can diffuse into the cell nucleus and binds to DNA proportionately to its amount; thus, by measuring the PI signal, the DNA content of each cell can be determined. Briefly, cells were seeded (2 × 10^6^ cells/well) in a 6-well microtiter plate followed by overnight incubation. The cells were synchronized by starvation in serum-free medium for 24 h. After appropriate treatment, the cells were detached and fixed with 70% ethanol for at least 2 h, before staining with PI solution (mixed with RNase) under dark condition for 20 min. The cells were then analyzed with a flow cytometer.

### 2.7. Cell Morphology

Morphological study is based on the identification of the leukaemia cell line and stage of cell differentiation. Jurkat, HL-60 and K-562 cells were treated with Thymoquinone. We used Hydrogen Peroxide (H_2_O_2_) as a positive control and ethanol as a negative control. Slides of the cell suspension were prepared by Cytospins 4 (Thermo Fisher Scientific, Waltham, MA, USA), first adding Bovine Albumin 22% specific, then centrifuge at 750 rpm for 5 min. Slides were air-dried, methanol fixed. Cell were stained with Giemsa stain reagents. Slides were dried and then mounted onto a coverslip. Stained cells were observed using a light microscope and the power was 60X.

### 2.8. Metabolites Extraction

Metabolites were extracted from both TQ-treated and untreated cells using a combination of methanol: acetonitrile: water at a ratio of (2:2:1 *v/v*). Ice-cold solvent in a volume of one mL was added to the cell supernatant, and the mixture was quickly vortexed for 30s and incubated for 1 h at −20 °C, followed by spin for 15 min at 13,000 rpm at 4 °C. The supernatant was removed, and the sample was dried in a vacuum concentrator. Further, dry extracts were reconstituted in 100 μL of acetonitrile: water (1:1, *v/v*), vortexed for 10 min and spin for 15 min at 13,000 rpm at 4 °C to remove insoluble debris. Finally, the resultant supernatants were taken for LC-MS/MS run [9,10].

### 2.9. HPLC Workflow

Ten µL of sample was injected into a HPLC column (Hypersail gold; 150 mm × 4.6 mm, 5 μm) with a flow rate of 0.2 mL/min. The mobile phase consisted of 0.1% Formic acid and 99.9% ACN formic acid (0.1%, *v/v*), using a linear gradient programme where the component of solution was changed from 5% B to 100% B over 90 min, at a constant flow rate of 0.2 mL/min (95% A from 5% to 30% over 72 min, 30% to 100% over 10 min, and kept at 100% for 5 min at a flow rate of 0.25 mL/min). The column temperature was maintained at 30 °C [9,10].

### 2.10. LC-MS/MS

A LTQ XL™ linear ion trap instrument (Thermo Fisher Scientific) was used for the quantification of metabolites. Full scan scope was chosen from 100 to 1000 *m/z*. The spray voltage was set at −3.0 kV. The capillary voltage was fixed at 4.0 V and the temperature was set at 270 °C. Nitrogen was used as a sheath gas and the flow rate was set at 40 arbitrary units. Further, helium was used as the buffer gas for the run [9,10].

### 2.11. Data Processing

The data obtained was processed using (XC-MS) data processing software. The raw MS data (raw files) were processed using XC-MS for feature detection, retention time correction and alignment. The parameters in XC-MS were set as follows: Cent-Wave settings for feature detection (Δ *m/z* = 30 ppm, minimum peak width = 10 s and maximum peak width = 120 s) and *m/zwid* = 0.25, min frac = 0.5, and bw = 10 for chromatogram alignment. After careful evaluation of retention time, alignment was shown not to be required. Isotopic peaks and adducts were detected using CAMERA. The precursor was matched with METLIN database at 20 ppm accuracy.

### 2.12. Multivariate Data Analysis

After data preprocessing, multivariate data analysis is then used to identify the metabolites that change most significantly because of treatments or over time. Different multivariate models are used in metabolomics, including principal component analysis (PCA), partial least squares discriminant analysis (PLS-DA), and orthogonal partial least squares discriminant analysis (OPLS-DA) as the most common methods.

### 2.13. Statistical Analysis

All the experiments were performed in triplicates. Statistical analyses were performed using the One-way ANOVA and *t*-test. Graphs were produced using GraphPad Prism v5.0 and the Microsoft Excel program.

## 3. Results

### 3.1. Effect of Thymoquinone on Viability of Cell Line

**Figure 1:**
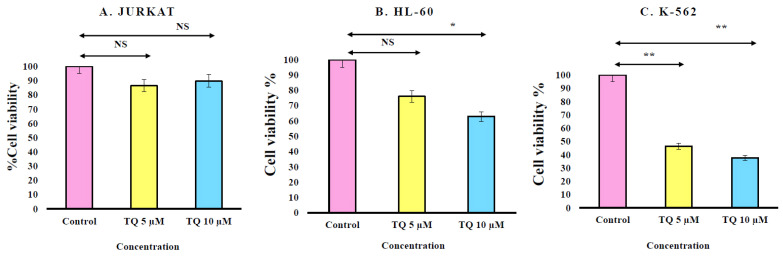
Cell viability was determined by using trypan blue exclusion test. Cells were cultured in the presence of different concentrations of TQ. TQ treated cell viability in (**A**) Jurkat, (**B**) HL-60 and (**C**) K-562 exposed for 24 h viability was determined and compared to untreated cells. Values are shown as mean ± S.E.M. Where * *p* < 0.05 and ** *p* < 0.01.

Cell viability was determined by the trypan blue exclusion test. Cells were cultured in the presence of different concentrations of TQ. TQ treatment of (A) Jurkat, (B) HL-60 and (C) K-562 showed a clear induction of trypan blue positive cells when compared to untreated controls. For statistical analysis, we used the one-way ANOVA test and data are shown as mean ± S.E.M (n = 12). Overall, we found that TQ treatment at both doses used in the current study significantly reduces the cell viability of both HL-60 and K-562 cells. However, we failed to see any significant reduction in Jurkat cell viability using trypan blue assay (Figure 1).

### 3.2. Effect of Thymoquinone on Cell Proliferation of Cell Line

**Figure 2:**
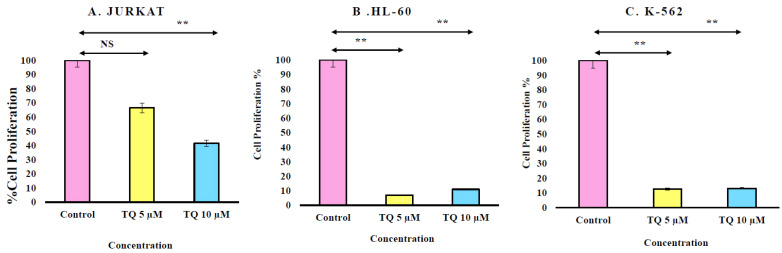
Cell growth was determined by WST-1 assay that measuring the growth rate on (**A**) Jurkat, (**B**) HL-60 and (**C**) K-562 cell. Cells were cultured in the presence of different concentrations of TQ. Value are shown as mean ± S.E.M. Values are shown as mean ± S.E.M. Where ** *p* < 0.01.

Cell proliferation was determined using the WST-1 assay, which measures the mitochondrial metabolism. Briefly, Jurkat, HL-60 and K-562 cell lines were cultured in the presence of different concentrations of TQ. For statistical analysis, we used the one-way ANOVA test and data are shown as mean ± S.E.M (n = 12). Data showed that in the Jurkat cell line, only high doses of TQ i.e., 10 µM, were able to reduce cell proliferation. However, TQ was able to reduce cell proliferation of both HL-60 and K-562 at both doses used in the current study. Overall, this data clears that Jurkat cells are less sensitive towards TQ treatment in comparison to HL-60 and K-562 (Figure 2).

### 3.3. Effect of Thymoquinone on Cell Death

**Figure 3:**
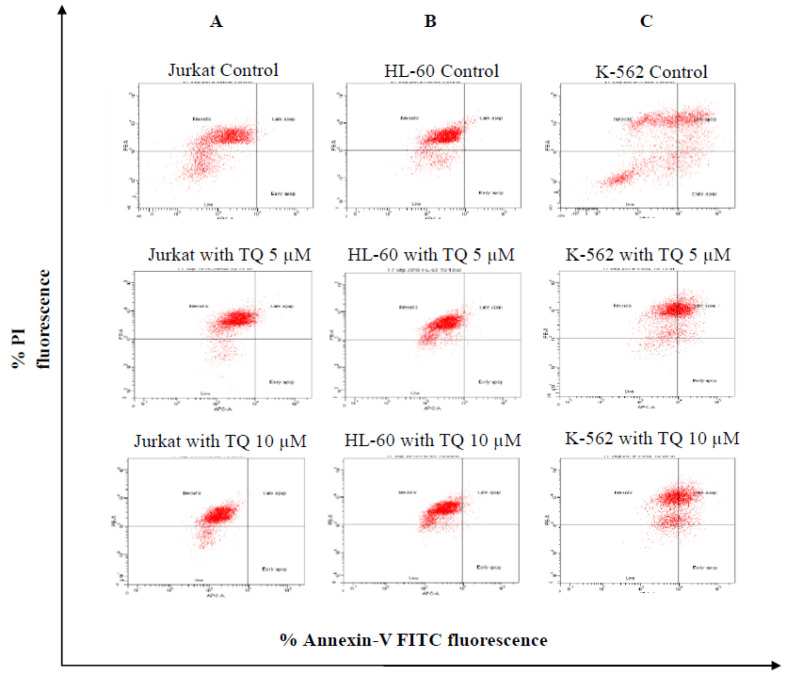
Representative dot blot of Annexin-V and PI on leukemic cells Data represented as dot blot where the lower left quadrant shows the live cells (Ann−, PI−), the lower right quadrant represents the early apoptotic cells (Ann+, PI−), the upper left quadrant represents necrotic cells (Ann−, PI+), and the upper right quadrant represents the late apoptotic cells (Ann+, PI+). Annexin-V and PI expression on Jurkat, HL-60 and K-562 cells as determined by flow cytometry.

Apoptosis assay was performed to assess cell death of cell lines during TQ treatment. Briefly, Jurkat, HL-60 and K-562 cells were treated with TQ for 24 h. Then, all leukemic cells were stained with Annexin-V and PI, and data was acquired by using flow cytometry to determine live, early apoptosis, late apoptosis and necrotic cells. Data in (Figure 3) represented the dot blot of expression, where the lower left quadrant represents the live cells (Ann−, PI−), the lower right quadrant represents early apoptotic cells (Ann+, PI−), the upper left quadrant represents necrotic cells (Ann−, PI+), and the upper right quadrant represents the late apoptotic cells (Ann+, PI+).

Results of apoptosis data showed that late apoptosis was induced by TQ in both HL-60 (2.1%) and K-562 (30.4%). However, TQ treatment fails to induce any significant levels of apoptosis in Jurkat cells (0.8%) (Supplementary Table S1).

### 3.4. Effect of Thymoquinone on Cell Cycle

**Figure 4:**
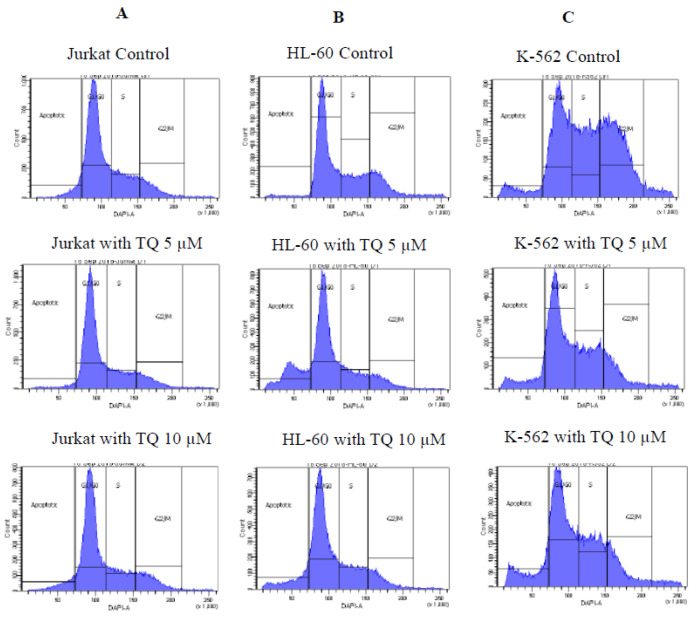
Histograms for cell cycle from analysis of (**A**) Jurkat, (**B**) HL-60 and (**C**) K-562 were treated with increasing concentration of TQ for 24 h. Cells were exposed to TQ, stained with PI and were analysed by using flow cytometry.

We determined the impact of TQ on Jurkat, HL-60 and K-562 cell cycles. Briefly, cells were cultured and treated with TQ for 24 h. Leukemic cells were further stained with PI, and results were acquired by using flow cytometry that determines apoptotic (sub-G1), G1/G0, S and G2/M features. The data of cell cycle were represented in histograms for the cell cycles. The results revealed that TQ treatment was blocking all of the three cell types in the G1/G0 phase of the cell cycle (Figure 4 and Supplementary Table S2), which is considered as the eukaryotic cell division phase.

### 3.5. Effect of Thymoquinone on Morphological Study

**Figure 5:**
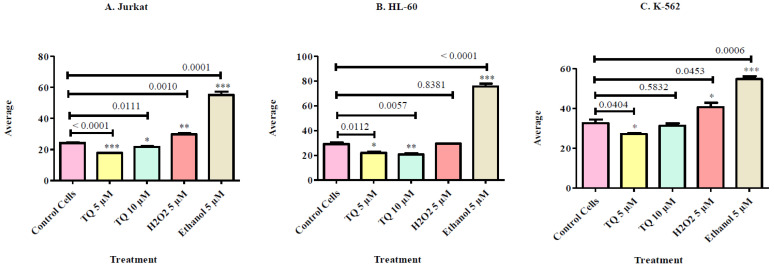
Different appearance of Leukemic cells Morphologic studies of leukemic cell line using the light microscope were carried out to observe the morphologic changes in Jurkat, HL-60 and K-562 cell line treated with TQ (5 µM and 10 µM) compare with un treated cells (control), positive control H_2_O_2_ and negative control after 24 h. The P-value for (**A**) Jurkat, (**B**) HL-60 and (**C**) K-562 measurement by one-way ANOVA and denoted as P; * ≤0.01, ** ≤0.005 and *** ≤0.0001. The figure showed P-value between control and TQ dose 5 µM, Control and 10 µM and control and positive and negative control by *t*-test analysis.

Morphologic studies of leukemic cell lines using the light microscope were carried out to observe the morphologic changes in Jurkat, HL-60 and K-562 cell lines, treated with TQ (5 µM and 10 µM), compared with untreated cells (control) after 24 h. Our results show that the size and number of cells were decreased compared to the untreated cells. The figure shows the decrease in length of the nucleus compared between control, treatment and positive and negative control. TQ treatment showed a significant decrease compared to the control cells (Figure 5 and Supplementary Figure S1).

### 3.6. Metabolomic Alterations Induced by TQ in Leukemic Cells

**Figure 6:**
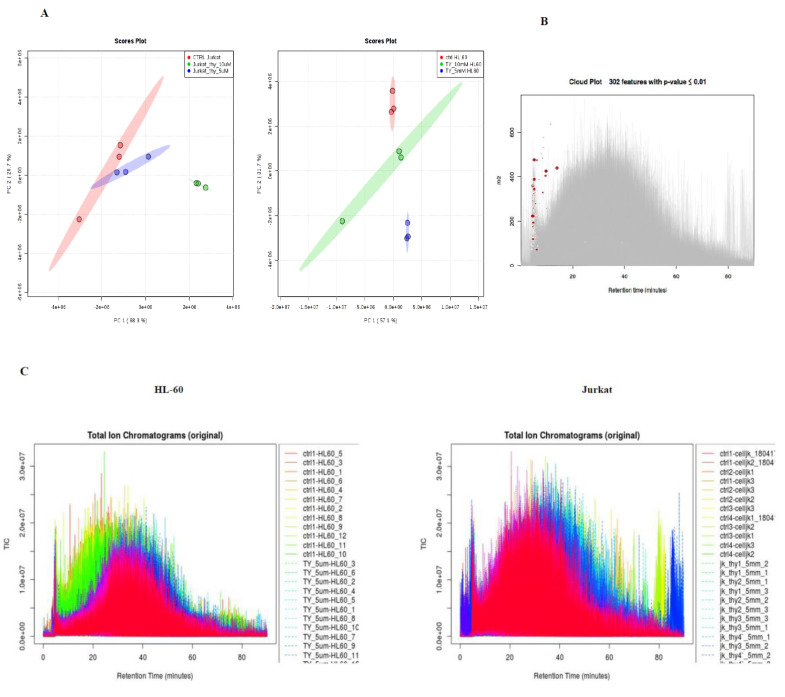

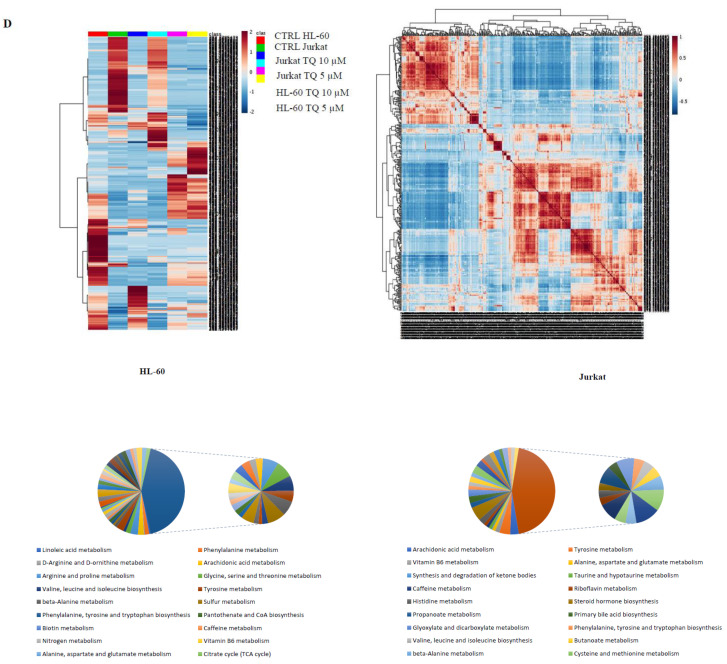
Metabolomics of TQ treated leukemic cell lines. (**A**) PCA analysis of six group it represents samples in the groups were closely cluster to one another. (**B**) Total Ion Chromatogram of all six groups and significant metabolic features are marked in respective retention time and spot size indicate its abundance. (**C**) Total Ion Chromatogram of all HL-60 and Jurkat cells. (**D**) Heatmap of pairwise correlation values of 356 metabolites along with depiction of major metabolic pathway of HL-60 and Jurkat cells affected by TQ. The Pearson correlation coefficients were calculated for log2-transformed ratios of the median values of CTRL with treated Jurkat and HL-60. For a better overview only, metabolites with the highest reproducibility (>95%) were displayed.

To explore the metabolic profile of HL-60 and Jurkat cells treated with TQ, we used an untargeted metabolomics approach using high performance liquid chromatography (HPLC) coupled to an electrospray ionization Linear ion trap (LTQ MS/MS). The spectrum of metabolites was acquired by using the Data Dependent Acquisition (DDA) method and the RAW files were processed. The PCA performed on all the samples clearly shows that all groups of samples were tightly clustered in the PCA score plot (Figure 6A). A total number of 335 features (metabolites) was identified in both cell lines. Among them, 302 features showed statistically significant (*p*-value ≤ 0.01) modulation in their levels (Figure 6B). The heat map implies that TQ has strong effects, resulting in metabolic variation on both of the cell lines used in the current study (Figure 6C). The list of altered metabolites showed metabolic alteration of different pathways (Table S1). The Major Metabolic pathway of Jurkat and HL-60 cell lines is shown in (Figure 6D and Supplementary Figures S2–S4).

(a) TQ modulates amino acid and lipid metabolism

**Figure 7:**
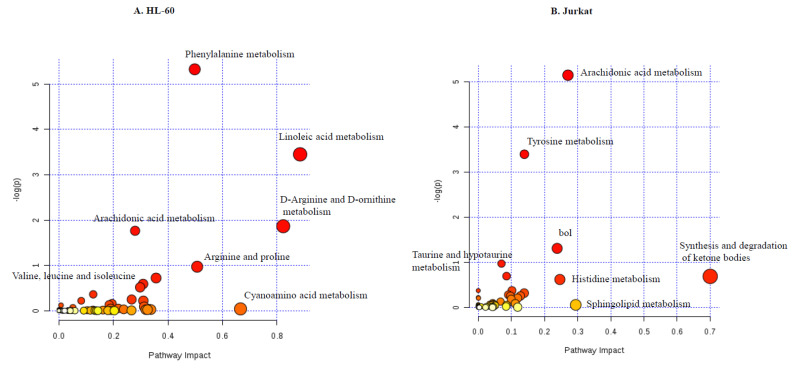
Identified pathways altered by TQ treatment in both (**A**) HL-60 and (**B**) Jurkat cell lines.

Pathway enrichment analysis, using a list of identified metabolites altered by TQ, showed dramatic alterations in many pathways. Metabolic pathways involved in apoptosis were observed to have a higher impact on TQ treatment. In HL-60, phenylalanine metabolism shows higher impact during TQ treatment. Phenylalanine activates Rho-associated kinase (ROCK), and it induces apoptosis. Tyrosine metabolism interlinked with other metabolic pathways like glutamine and tyrosine kinase, which is downstream activation of apoptosis, shows higher impact on TQ treatment. Other metabolic pathways of the vitamin B6 metabolism, which enable the sensitization of cancer cells to apoptosis, also showed alterations (Figure 7A). We found some heterogeneity between two cell types, but some metabolites are regulated in a common way by TQ treatment.

**Figure 8:**
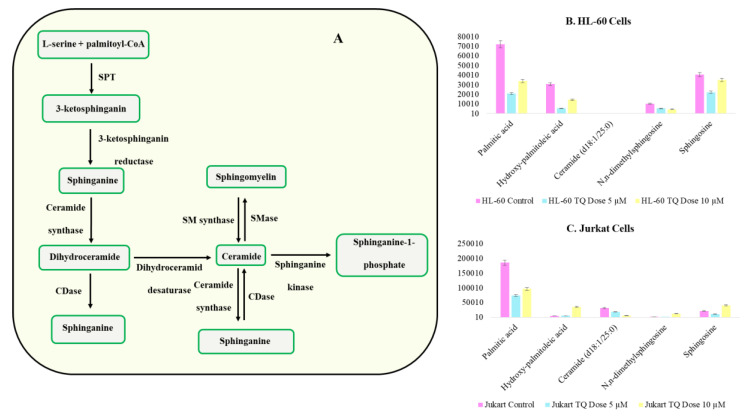
Lipids specifically sphingosine and ceramide pathway are key targets of TQ (**A**) The synthesis of sphingosine and ceramide started in the presence of serine palmitoyltransferase (SPT), a rate limiting enzyme of the pathway. (**B**) Metabolites related to lipid metabolism altered by TQ treatment in HL-60 cell line. (**C**) Metabolites related to lipid metabolism altered by TQ treatment in Jurkat cell lines.

Lipogenesis contributes to cancer through multiple cell regulatory mechanisms. It is important in fatty acid synthesis and rapidly combines into neutral and phospholipid stores; fatty acid derivatives from lipolysis can also assist as pioneers for important signaling lipids. TQ treatment reduces the accumulation of linoleic acid in both HL-60 and Jurkat cells (Supplementary Tables S2–S4). Linoleic acid metabolism alters cell proliferation and induces apoptosis, showing a higher impact on TQ treatment. Further, we observed alteration in arachidonic acid metabolism by TQ treatment (Figure 7B). We also found alteration in palmitic acid that plays a significant role in cell survival apoptosis. Palmitic acid is first converted into ceramide and then to sphingosine. Ceramide is toxic to cells and leads to apoptosis, and sphingosine is involved in cell survival. Palmitic acid levels were reduced significantly in both cell lines during TQ treatment. Further, ceramide levels were increased dramatically in both cell lines during TQ treatment (Figure 8A–C).

(b) TQ treatment alters TCA and amino acid metabolism

**Figure 9:**
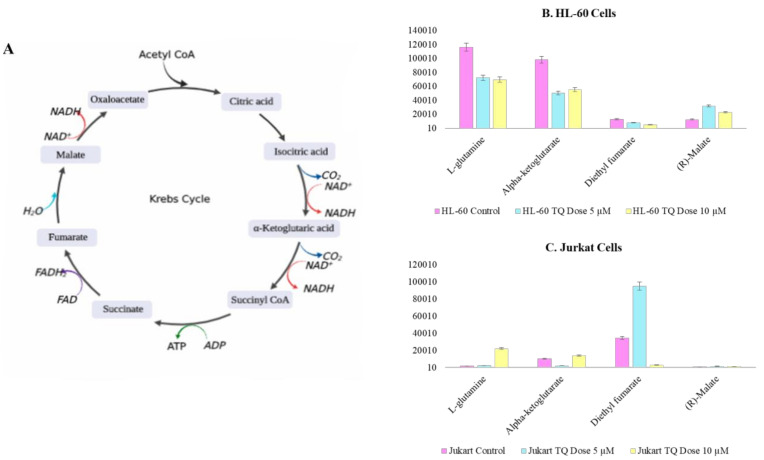
TQ alters TCA cycle (**A**) TCA cycle and cellular metabolism. (**B**) Variation between TCA metabolite observed due to effect of TQ treatment on HL-60 cells. (**C**) Variation between TCA metabolite observed due to effect of TQ treatment on Jurkat cells.

Thymoquinone (TQ) alters the metabolites involved in the TCA cycle. Glutamine is a key metabolite; it converts to alpha ketoglutarate (α-KG) and enters the TCA cycle. During TQ treatment, the HL-60 cells’ glutamine showed significantly reduced accumulation, but, in Jurkat, significant changes were not observed. α-KG is acceleration of cell proliferation, showed 2.0-fold and 8.0 reduction, while HL-60 and Jurkat cells were treated with a 5 µM concentration of TQ. A 2.4- and 11-fold reduction in the accumulation of oncometabolite diethyl fumarate was observed, while HL-60 and Jurkat cells showed a slightly increased accumulation when treated with a 5 µM concentration of TQ (Figure 9A–C).

(c) TQ alters metabolite involved in methylation reactions

**Figure 10:**
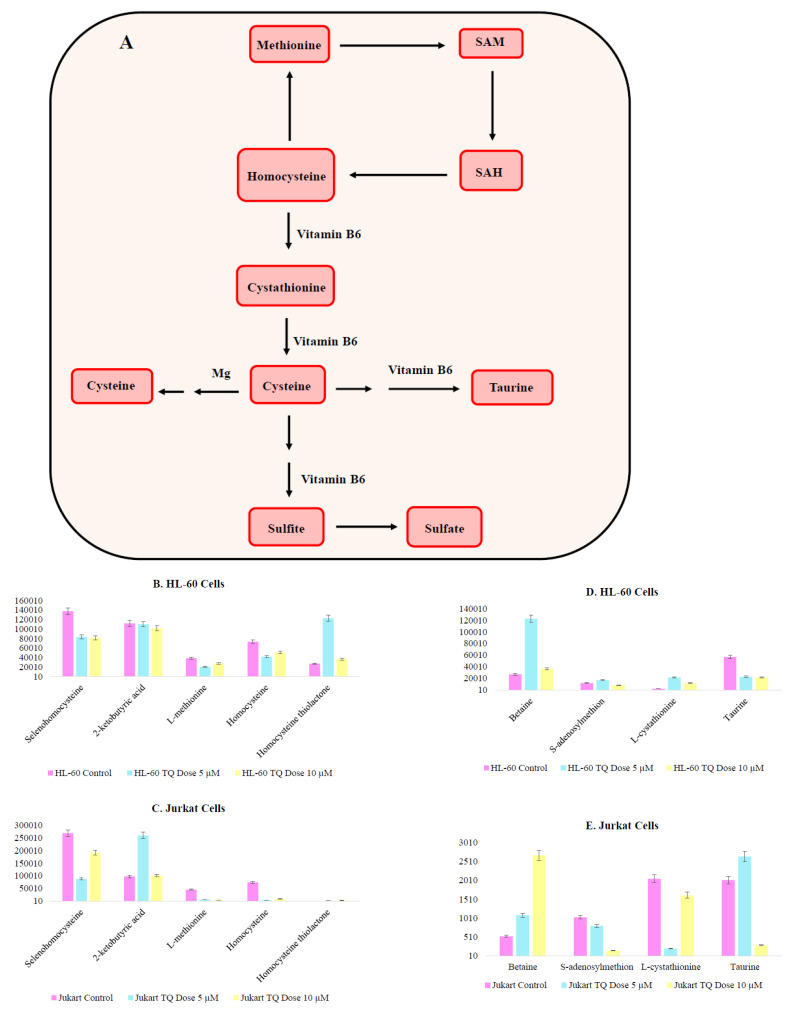
TQ treatment modulates epigenetic regulatory metabolite levels (**A**) DNA methylation pathway, where SAM is S-Adenosylmethionine and SAH is S-Adenosylhomocysteine (**B**–**E**) Metabolite variation in DNA methylation pathway.

**Figure 11:**
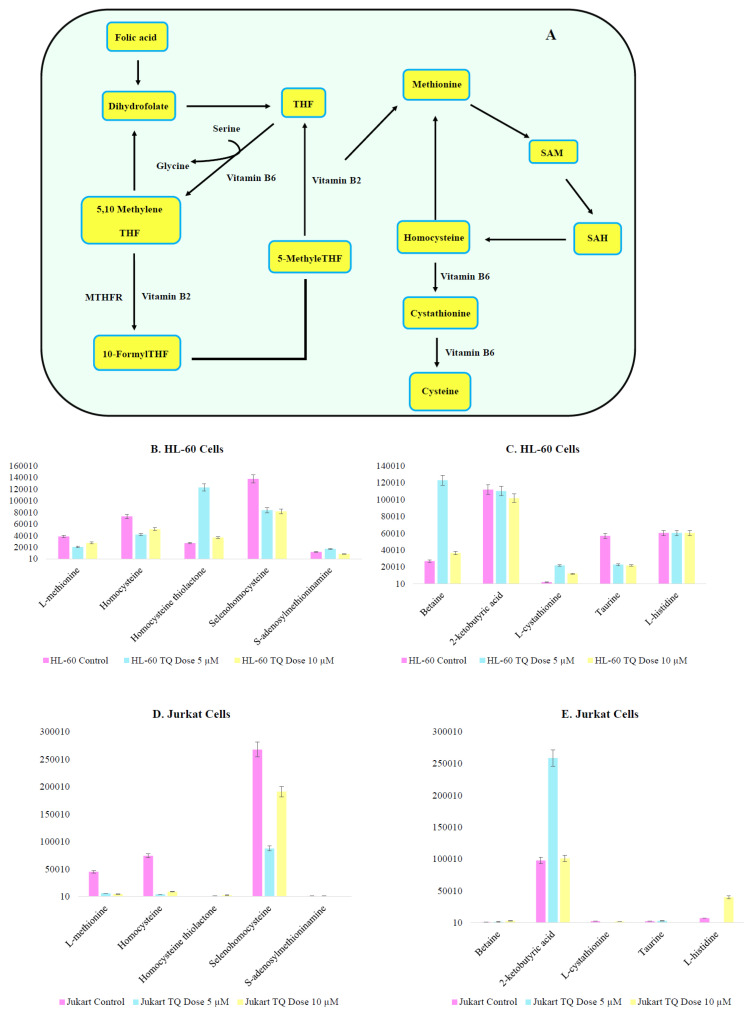
TQ alters metabolite levels associated with one carbon metabolism (**A**) One carbon Metabolism pathway, where THF is Tetrahydrofolate. (**B**,**C**) Metabolites related to one carbon metabolism altered by TQ treatment in HL-60 cell line. (**D**,**E**) Metabolites related to one carbon metabolism altered by TQ treatment in Jurkat cell lines.

Aberrant methylation in the promoters of gene coding for proteins implicated in the extrinsic pathway could be responsible for early blocking events in apoptosis. Both cysteine and methionine metabolism play a prominent role in DNA methylation (Figure 10A). TQ treatment has a high impact on metabolite responsible for DNA methylation and epigenetic medication. Metabolite homocysteine and L-methionine observed reduced accumulation on TQ treatment on both cell lines (Figure 10B,C). 6-Methyladenine and Methyl guanine show a decrease in their presence, which supports the TQ interference in cysteine and methionine metabolism. There was heterogeneity between HL-60 and Jurkat cells, but TQ interfered with the epigenetic modification of both cell lines (Table S1). Other metabolites like cystathionine, taurine and betaine are intermediaries that are involved in the DNA methylation process, and showed heterogeneous variation between two cell lines (Figure 10D,E). In addition to this we have found TQ modulates multiple metabolites of one carbon metabolic pathway (Figure 11A–E), further confirms that TQ have epigenetic modifying impact through modulation of metabolism.

## 4. Discussion

One of the earliest and most common methods for measuring cell viability is the trypan blue (TB) exclusion assay. Trypan blue binds to intracellular proteins, thereby rendering the cells a bluish color. The trypan blue exclusion assay allows for a direct identification and enumeration of live (unstained) and dead (blue) cells in a given population. However; it is not able to differentiate between apoptotic and necrotic cells. In the present study, Jurkat, HL-60 and K-562 were cultured in RPMI-1640 medium and treated with Thymoquinone for 24 h. TQ inhibited cell viability in leukemic cells, and the more significant cells that were affected by TQ compounds were K-562 cells.

Further analysis of leukemic cells in cell proliferation of was determined by WST-1. The WST-1 assay is based on the cleavage of water-soluble tetrazolium salt-1, which is reduced extra-cellularly to formazan dye by enzymes of the plasma membrane oxidoreductase. The primary reductant is NADH, derived from the TCA of the mitochondria. WST-1 is converted by metabolically active cells and was employed to measure cell proliferation. The optical density of the formazan dye is measured to get an estimate of live cells. In the present study, Jurkat, HL-60 and K-562 was cultured in RPMI-1640 medium and treated with Thymoquinone for 24 h. TQ treatment is significant on HL-60 and more significant on K-562 cells.

The level of cell death in cancer cells was assessed with the Annexin V-PI kit. Annexin V can specifically bind to phosphatidylserine. Cells undergoing cell death will experience diffusion of phosphatidylserine from inner cell membrane to outer cell membrane, whereby these proteins can be quantified by targeting them with fluorescence-tagged Annexin V. The necrosis as a form of cell death is almost always associated with a pathological process. We showed in (Table S1) that cells die by necrosis more than apoptosis on Jurkat, HL-60 and K-562 after treatment by TQ.

Cell cycle progression of leukemia cells was examined by flow cytometry after propidium iodide (PI) staining. Cell cycle phases can be differentiated by measuring their DNA content, where G1 phase is 2n, S phase is between 2n and 4n, and G2 phase is 4n. PI can diffuse into the cell nucleus and binds to DNA proportionately to its amount. Jurkat, HL-60 and K-562 cells were stained with DNA-specific fluorochrome Propidium iodide. TQ interferes with DNA structure. It aims at cellular copper, which is current in the chromatin and is closely associated with DNA base guanine, and is the reason for oxidative breakage to DNA and consequent cancer cell death. In an earlier study, TQ was found to inhibit DNA synthesis, proliferation, and viability of cancerous cells, such as LNCaP, C4-B, DU145, and PC-3, but not noncancerous BPH-1 prostate epithelial cells [5]. We showed in (Table S2) that DNA content is more in G1/G0 phases on Jurkat, HL-60 and K-562 after treatment by TQ.

The morphological variation between untreated and treated leukemic cells correlated to their different types of cells and treatments. In the present study, Jurkat, HL-60 and K-562 were cultured in RPMI-1640 medium and treated with TQ for indicated time. Hydrogen Peroxide (H_2_O_2_) as a positive control and ethanol as a negative control for 24 h. On the second day, treated and untreated leukemic cell lines was prepared by Cytospins 4, cells were stained with Giemsa stain reagents and then cells were observed using a light microscope and the power was 60X. TQ treatment showed significant decrease in cell size when compared the control cells treated with H_2_O_2_ and Ethanol.

In the current work, we provided a comprehensive map of metabolites and metabolic pathways altered by TQ in cancer cells. We found that TQ alters major metabolic pathways like TCA cycle, amino acid metabolism, lipid metabolism and metabolites important for methylation-related events.

We decided to use an active ingredient extracted from natural products or traditional medicine because these medicines have been used in the human body for many generations, and any possible adverse effects/toxicities have been well identified and are likely manageable. Cancer cell lines are well established models to study specific cellular mechanisms characteristic of different types of cancer, usually by monitoring specific proteins and their actions [11]. Thus, in this project, we focused on the anticancer activities of thymoquinone, a natural product isolated from *Nigella sativa*, in the treatment of leukemia, rather than chemotherapy and radiotherapy, which targets cancerous and healthy cells, and tried to elucidate its mechanism of action in multiple signaling pathways. Resent advances in omics technologies allow for more systematic investigation to characterize parameters like gene expression, protein and metabolite profiles. For the application of omics technologies to the understanding of compound action, it is essential that the effects of compounds produce a sensitive response, which can be reproduced with sufficiently high quality. Here, we have shown that the metabolic profiling of cancer cells can not only identify phenotypic differences between cell lines, but can also sensitively detect and distinguish changes in metabolite concentrations induced by relatively short exposure to differing compounds. It is important to mention that all treatments caused a distinct reproducible profile, which is observed in unsupervised PCA. TQ alters the metabolic signature of leukemic cells, and earlier it showed that TQ activates ROS production and p38 phosphorylation in breast cancer cells. This results in TQ’s effects in anti-proliferative and pro-apoptotic effects. Metabolic pathways play a key role in cellular physiology. TQ alters major metabolic pathways, which results in induction of apoptosis and disturbances in cellular homeostasis. Even though there is heterogeneity between the two cell lines, TQ activates some similar metabolite changes, which results in apoptosis and epigenetic variation.

It has been shown that TQ can induce DNA damage in glioblastoma cells [12]. Here, we found significant accumulation of thymine glycol, an intermediary metabolite known to induce DNA damage and apoptosis in cancer cells [13]. Therefore, we believe that TQ promotes the accumulation of thymine glycol to promote DNA damage of leukemic cells, to induce apoptosis. However, this assumption needs further scientific evidence. Further, we observed a strong reduction in cellular guanine pools during TQ treatment. High guanine levels are maintained by cancer cells to facilitate nucleotide metabolism and their sufficient pools [14,15,16].

We found that α-KG is acceleration of cell proliferation, which shows decreases in its fold variation irrespective of two cell lines and doses. It was also shown to be a reduced accumulation of onco-metabolites like Fumarate. The palmitic acid pathway is a key regulatory element in cell survival apoptosis. Palmitic acid converts into ceramide, and then to sphingosine. Two major metabolites involved in palmitic acid pathways are ceramide and sphingosine, which are involved in cell toxicity and cell survival. Significantly, ceramide shows an increase in its accumulation, which supports the TQ-induced apoptosis. TQ also has major effects on DNA methylation metabolites.

## 5. Conclusions

TQ alters the metabolome of human leukemic cells. We have demonstrated that the metabolites involved in anti-proliferative and pro-apoptotic pathways are modulated by TQ treatment and might be involved in apoptosis and epigenetic modifications. We strongly believe that our results globally demonstrate the metabolic effect of TQ in leukemic cell lines and provide clues for the identification of novel metabolic targets of TQ in cancer cells.

## Supplementary Materials

The following are available online at https://www.mdpi.com/2072-6643/12/6/1792/s1, Figure S1: Different morphological size of Leukemic cells, Figure S2: 2D Variation of Difference between RAW files in HL-60 Cell and Jurkat Cell, Figure S3: Major Metabolic Changes by Thymoquinone, Figure S4: Structure of the some compound founded in metabolism with the important, Table S1: Percentages of live, early apoptotic, late apoptotic, and necrotic leukemia cells upon treatment with TQ treatment by flow cytometry, Table S2: The percentages of the cell cycle phases of leukemia cells line upon treatment with TQ treatment by flow cytometry. Table S3: Raw metabolomic datasets extracted from XCMS database, Table S4: Identification of metabolic pathways associated by Thymoquinone treatment in (A) HL-60 and (B) Jurkat.
