# Supplementary material for: Untargeted Metabolomics Identifies Key Metabolic Pathways Altered by Thymoquinone in Leukemic Cancer Cells
Source: Nutrients. 2020 Jun 17;12(6):1792. doi: 10.3390/nu12061792 (PMC7353463; doi:10.3390/nu12061792)
Supplement: Supplementary file 1 [file nutrients-12-01792-s001.pdf]

**Supplementary Figure 1.** Different morphological size of Leukemic cells.

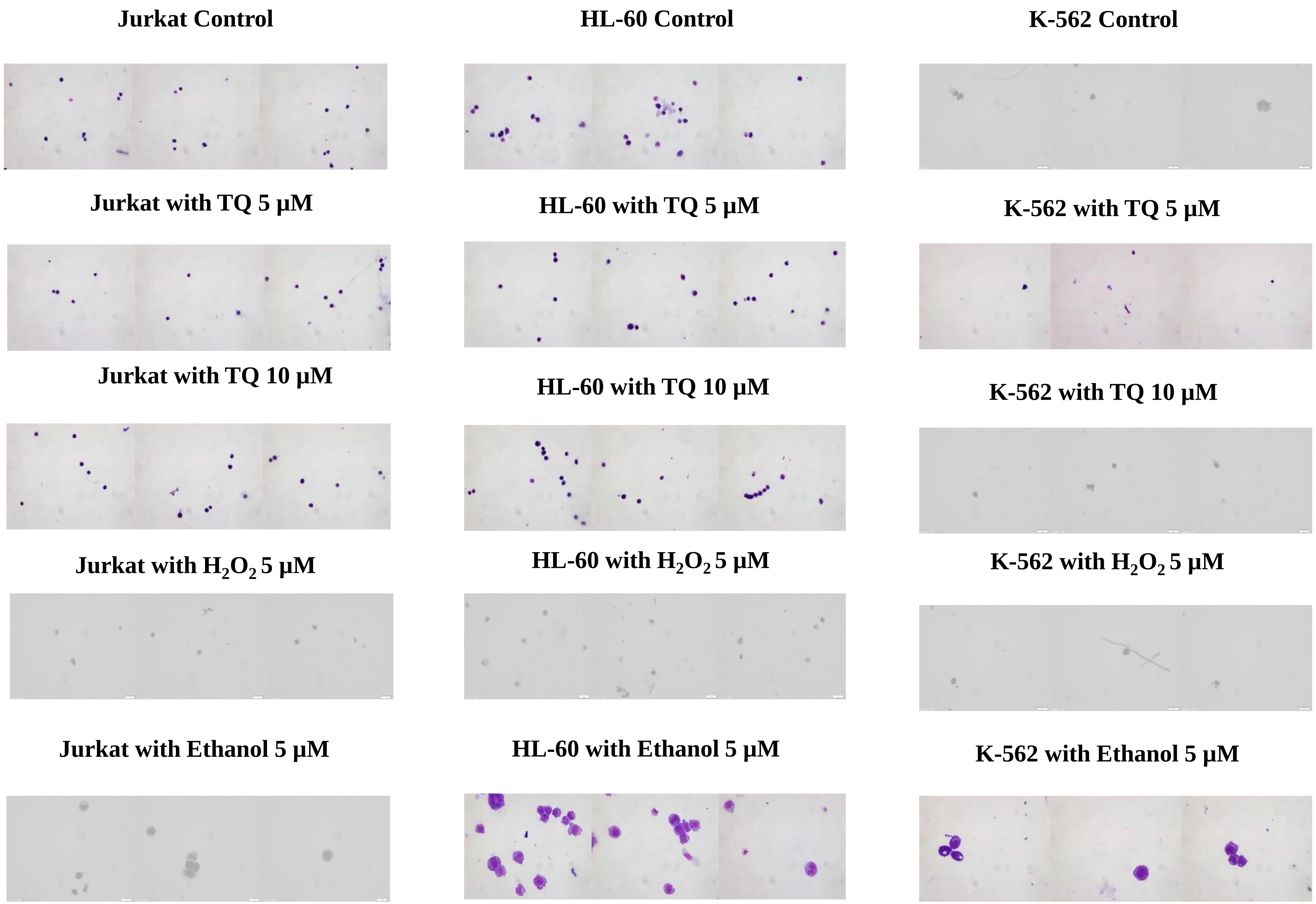

**Supplementary Figure 2.** 2D Variation of Difference between RAW files in HL-60 Cell and Jurkat Cell.

**HL-60**

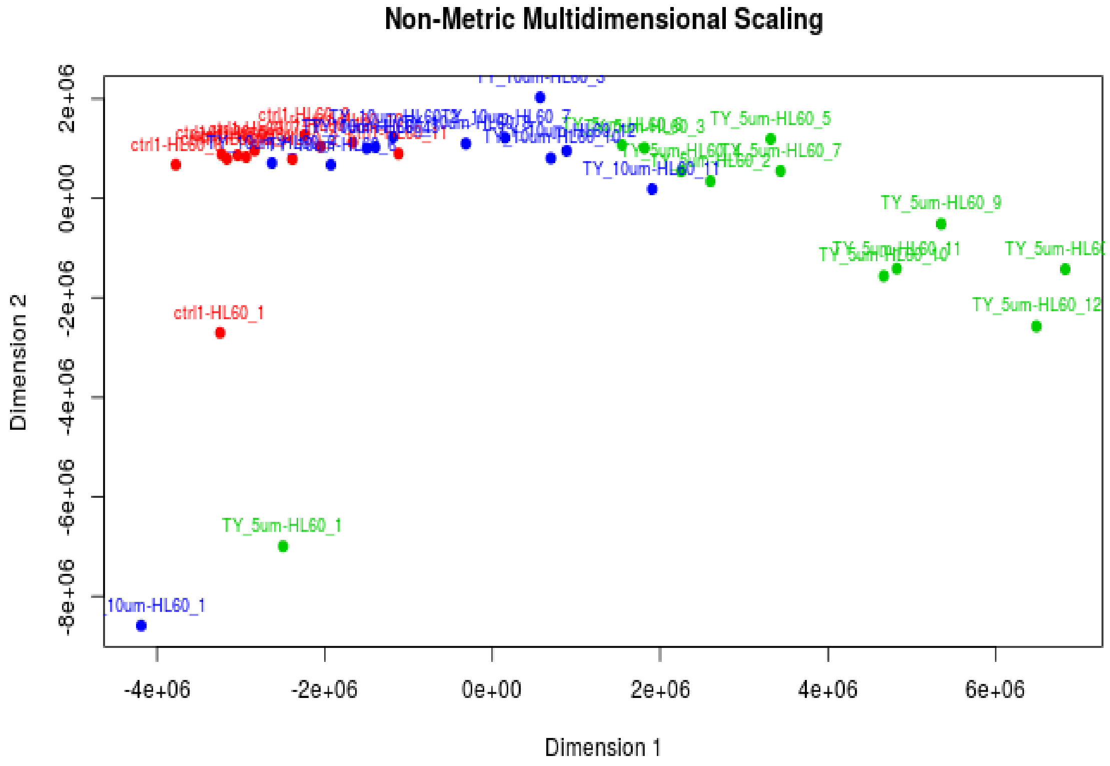


**Jurkat**

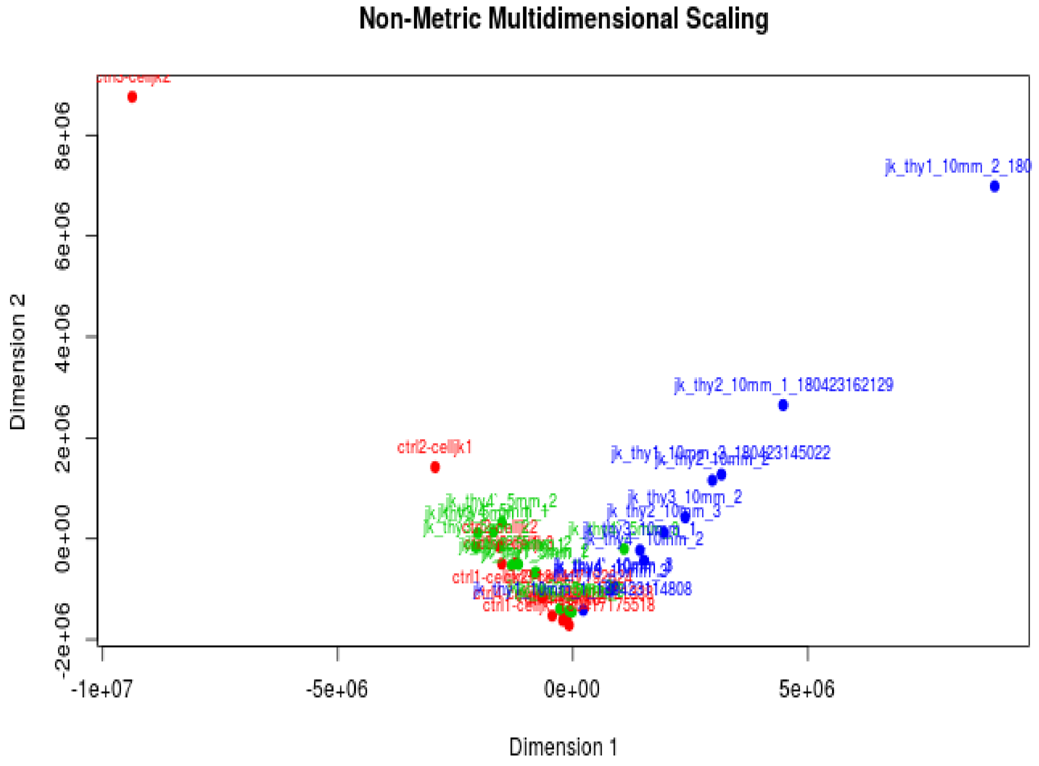

**Supplementary Figure 3.** Major Metabolic Changes by Thymoquinone.

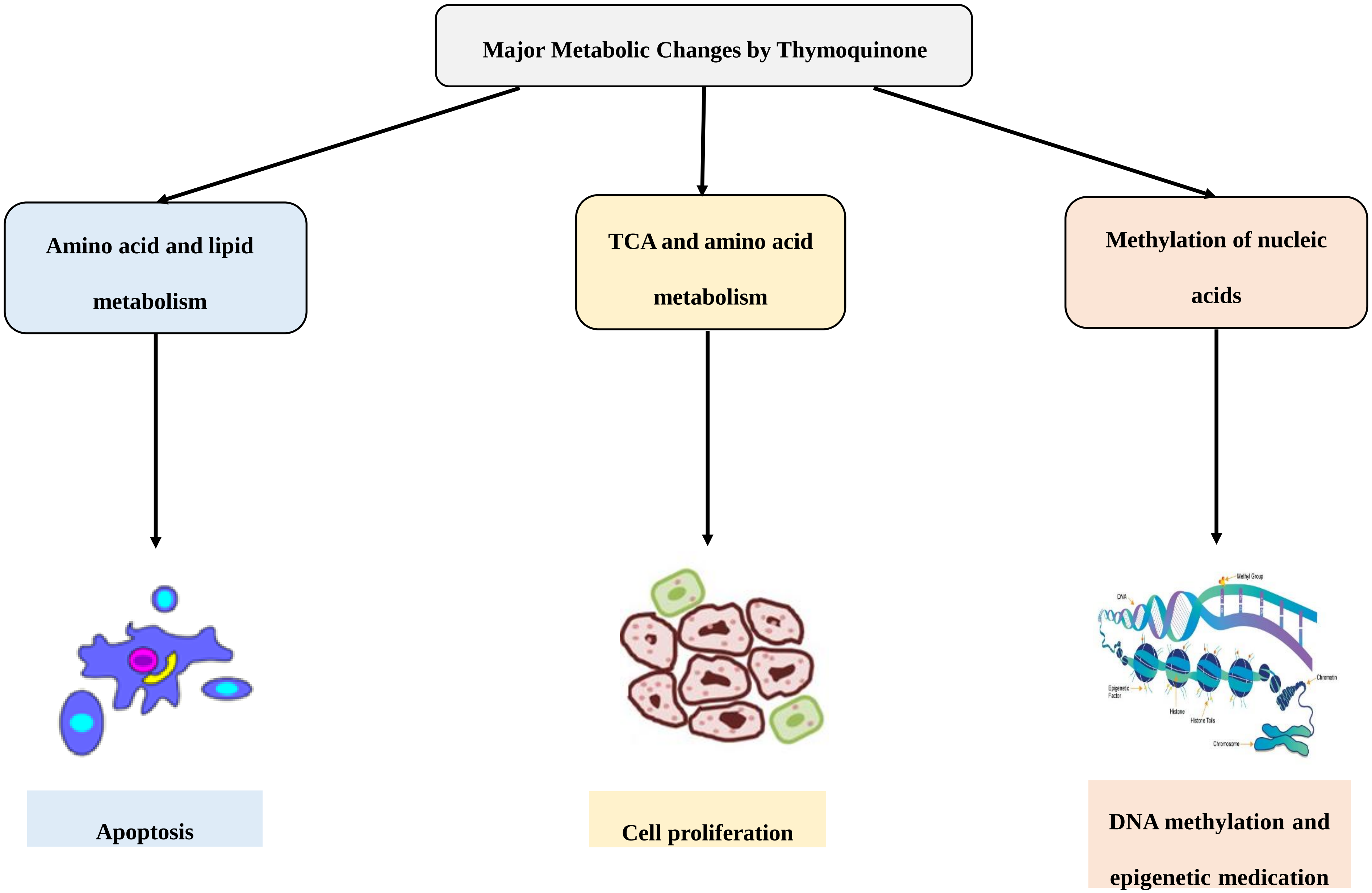

**Supplementary Figure 4.** Structure of the some compound founded in metabolism with  the important.

**A. 6-methyladenine**

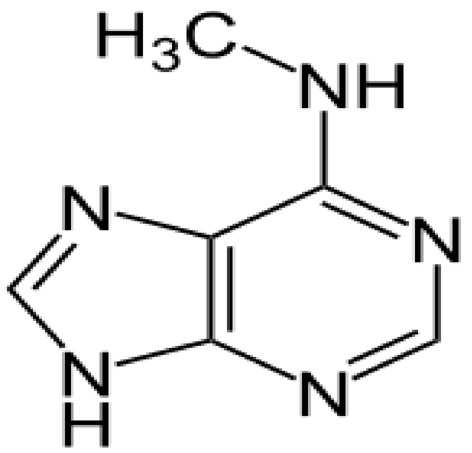


**B. Thymine glycol**

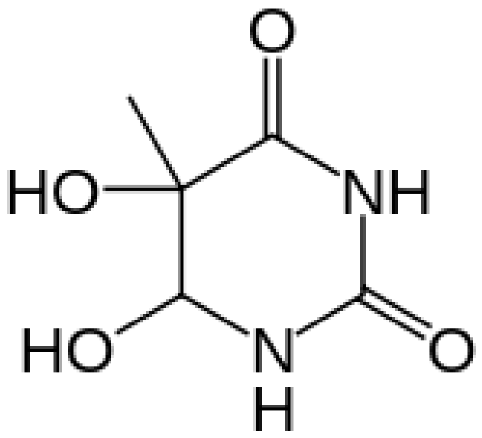


**C. Perillic acid**

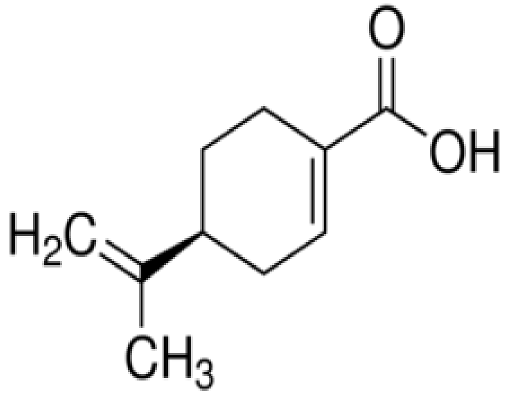


**(A)** Methylation of N6 adenosine (m6A) is the most abundant form of messenger RNA (mRNA) modification in eukaryotes. It is known to play crucial roles in the regulation of gene expression, protein translation, and splicing in normal biology.

**(B)** The presence of thymine glycol in DNA can have profound consequences on DNA replication. The presence of thymine glycol may also induce structural changes at the initiation and termination points leading to alterations in the relative amounts of the termination bands of DNA synthesis.

**(C)** Perillic acid is currently on clinical trials as a multi-target anti-cancer agent. The prospective anticancer activity of perillic acid relates to the accepted mechanism for these monoterpenes as inhibitors of protein prenylation in cancer cells.

**Supplementary Table 1.** Percentages of live, early apoptotic, late apoptotic, and necrotic leukemia cells upon treatment with TQ treatment by flow cytometry.

| Jurkat cell | | | |
|---|---|---|---|
| **Phases** | **Control** | **TQ Dose 5 µM** | **TQ Dose 10 µM** |
| **% Necrotic** | 75.1 | 90.2 | 89.3 |
| **% Late Apoptosis** | 0.8 | 3.3 | 0 |
| **% Live** | 24.1 | 6.5 | 10.7 |
| **% Early Apoptosis** | 0 | 0 | 0 |
| **HL-60** | | | |
| **Phases** | **Control** | **TQ Dose 5 µM** | **TQ Dose 10 µM** |
| **% Necrotic** | 85.7 | 90.4 | 90.9 |
| **% Late Apoptosis** | 2.1 | 2.5 | 2.8 |
| **% Live** | 12.1 | 7 | 6.1 |
| **% Early Apoptosis** | 0 | 0.1 | 0.2 |
| **K-562 cell** | | | |
| **Phases** | **Control** | **TQ Dose 5 µM** | **TQ Dose 10 µM** |
| **% Necrotic** | 33.4 | 50.3 | 51.4 |
| **% Late Apoptosis** | 30.4 | 42 | 39.3 |
| **% Live** | 29.6 | 6.1 | 6.2 |
| **% Early Apoptosis** | 6.6 | 1.6 | 2.5 |

**Supplementary Table 2.** The percentages of the cell cycle phases of leukemia cells line upon treatment with TQ treatment by flow cytometry.

| Jurkat cell | | | |
|---|---|---|---|
| **Cell cycle phases** | **Control** | **TQ Dose 5 µM** | **TQ Dose 10 µM** |
| **Apoptotic** | 6.6 | 3.2 | 7.3 |
| **G1/G0** | 61.1 | 64 | 59.2 |
| **S** | 20.3 | 19.4 | 18.8 |
| **G2/M** | 11 | 12 | 13.9 |
| **HL-60 cell** | | | |
| **Cell cycle phases** | **Control** | **TQ Dose 5 µM** | **TQ Dose 10 µM** |
| **Apoptotic** | 2.7 | 21.9 | 12.2 |
| **G1/G0** | 55.3 | 53.1 | 57.5 |
| **S** | 21.7 | 15.1 | 18.9 |
| **G2/M** | 17.7 | 9.4 | 10.6 |
| **K-562 cell** | | | |
| **Cell cycle phases** | **Control** | **TQ Dose 5 µM** | **TQ Dose 10 µM** |
| **Apoptotic** | 4.8 | 9.8 | 13.4 |
| **G1/G0** | 31.1 | 47.5 | 45.4 |
| **S** | 27.3 | 25.8 | 25.2 |
| **G2/M** | 33.3 | 14.6 | 13.6 |

**Supplementary Table 3.** Raw metabolomic datasets extracted from XCMS database.

| featureidx | name | mzmed | mzmin | mzmax | rtmed | rtmin | rtmax | npeaks |
|---|---|---|---|---|---|---|---|---|
| 1 | M86T5 | 86.03821 | 85.92044 | 86.05632 | 4.632783 | 3.464748 | 4.884887 | 19 |
| 2 | M403T8 | 403.3552 | 403.337 | 403.3719 | 8.345605 | 8.087223 | 8.549472 | 10 |
| 3 | M344T5 | 344.4143 | 344.3549 | 344.4491 | 5.165696 | 4.825887 | 5.835453 | 12 |
| 4 | M388T5 | 388.4465 | 388.4392 | 388.5077 | 5.171168 | 4.817698 | 5.495433 | 12 |
| 5 | M294T5 | 294.2631 | 294.2308 | 294.321 | 4.579524 | 4.534977 | 4.722842 | 10 |
| 6 | M323T5 | 323.2353 | 323.1773 | 323.2531 | 4.910777 | 4.748247 | 5.22985 | 21 |
| 7 | M404T8 | 404.3817 | 404.3616 | 404.4263 | 8.352753 | 8.089535 | 8.489643 | 10 |
| 8 | M314T5 | 314.2733 | 314.2556 | 314.324 | 4.59147 | 4.563005 | 4.728507 | 15 |
| 9 | M127T5 | 127.0584 | 126.9882 | 127.1454 | 4.635805 | 4.592405 | 5.175502 | 22 |
| 10 | M174T5 | 174.0514 | 174.0011 | 174.1457 | 4.603803 | 3.766963 | 4.938027 | 32 |
| 11 | M311T5 | 311.2684 | 311.2631 | 311.318 | 4.710884 | 4.615372 | 4.883565 | 10 |
| 12 | M132T5 | 132.056 | 131.9632 | 132.1308 | 4.626476 | 4.404047 | 4.975167 | 30 |
| 13 | M477T5 | 476.5485 | 476.4845 | 476.5636 | 5.170188 | 4.800562 | 5.644097 | 13 |
| 14 | M133T5 | 133.0708 | 133.0053 | 133.1309 | 4.597893 | 4.52215 | 4.807623 | 22 |
| 15 | M125T5 | 125.0504 | 125.0114 | 125.1242 | 4.689032 | 4.55158 | 5.354145 | 23 |
| 16 | M297T5 | 297.1961 | 297.1628 | 297.2427 | 4.582013 | 4.534977 | 4.677878 | 17 |
| 17 | M325T5 | 325.3278 | 325.2711 | 325.3775 | 4.839259 | 4.631947 | 5.094492 | 22 |
| 18 | M331T6 | 331.3559 | 331.2392 | 331.432 | 5.61722 | 5.301902 | 6.717672 | 23 |
| 19 | M359T5 | 359.3617 | 359.3464 | 359.5293 | 4.634463 | 4.565832 | 5.554575 | 14 |
| 20 | M270T7 | 270.2839 | 270.267 | 270.3285 | 6.967975 | 6.459652 | 7.178153 | 8 |
| 21 | M503T8 | 502.5382 | 502.5174 | 502.5491 | 7.872158 | 7.365097 | 8.207987 | 8 |
| 22 | M223T5 | 223.1618 | 223.0626 | 223.256 | 4.576538 | 4.510578 | 4.690018 | 12 |
| 23 | M120T5 | 120.0586 | 120.034 | 120.1237 | 4.683622 | 4.6082 | 4.95588 | 23 |
| 24 | M273T5 | 273.2951 | 273.2486 | 273.3382 | 4.87758 | 4.512252 | 5.282557 | 40 |
| 25 | M259T5 | 259.2548 | 259.2178 | 259.3301 | 4.833018 | 4.606828 | 5.226058 | 38 |
| 26 | M331T5 | 331.366 | 331.3329 | 331.4465 | 4.730055 | 4.563005 | 5.142007 | 25 |
| 27 | M130T5 | 130.0326 | 129.9596 | 130.1386 | 4.655774 | 3.583923 | 5.051045 | 32 |
| 28 | M276T5 | 276.254 | 276.1973 | 276.2711 | 4.64802 | 4.623633 | 4.910857 | 17 |

| | | | | | | | | |
|---|---|---|---|---|---|---|---|---|
| 29 | M430T5 | 430.4428 | 430.3497 | 430.4803 | 4.705287 | 3.80082 | 5.594457 | 34 |
| 30 | M439T14 | 438.5027 | 438.4712 | 438.5394 | 13.92583 | 13.71556 | 14.09794 | 10 |
| 31 | M455T12 | 454.5221 | 454.4628 | 454.535 | 12.22604 | 12.05158 | 12.30137 | 10 |
| 32 | M295T5 | 295.2795 | 295.2559 | 295.3263 | 4.656998 | 4.615372 | 4.892948 | 7 |
| 33 | M88T5_1 | 88.05086 | 87.98825 | 88.15427 | 4.613634 | 4.024837 | 4.835405 | 28 |
| 34 | M456T12 | 455.5417 | 455.5253 | 455.5523 | 12.21907 | 12.04494 | 12.3036 | 9 |
| 35 | M173T5 | 172.9463 | 172.877 | 173.0582 | 4.600171 | 4.506617 | 5.058922 | 28 |
| 36 | M239T7 | 239.2435 | 239.1675 | 239.2718 | 7.287099 | 6.771642 | 8.004913 | 20 |
| 37 | M440T14 | 439.5411 | 439.4924 | 439.5525 | 13.92473 | 13.68874 | 14.07218 | 9 |
| 38 | M166T5 | 166.0695 | 166.0523 | 166.1781 | 4.744203 | 4.586708 | 5.381615 | 32 |
| 39 | M205T5 | 205.1406 | 205.0951 | 205.1868 | 4.768325 | 4.616197 | 5.08235 | 31 |
| 40 | M327T5 | 327.3658 | 327.2272 | 327.4204 | 5.18684 | 4.840658 | 5.737203 | 12 |
| 41 | M150T5 | 150.0651 | 150.0059 | 150.1196 | 4.584138 | 3.80082 | 4.763325 | 18 |
| 42 | M481T13 | 480.5497 | 480.5258 | 480.5534 | 12.82689 | 12.60807 | 12.94736 | 8 |
| 43 | M213T5 | 213.2154 | 213.1657 | 213.2302 | 5.209492 | 5.149608 | 5.598225 | 8 |
| 44 | M638T11 | 637.5551 | 637.534 | 637.6099 | 11.49579 | 10.94977 | 11.97891 | 9 |
| 45 | M115T5 | 114.9886 | 114.9373 | 115.15 | 4.681278 | 3.774872 | 6.00717 | 40 |
| 46 | M267T7 | 267.1999 | 267.1409 | 267.3027 | 6.855823 | 6.587305 | 7.48176 | 8 |
| 47 | M392T5 | 392.3849 | 392.3629 | 392.4408 | 4.68207 | 4.624823 | 4.82991 | 12 |
| 48 | M358T5 | 358.4234 | 358.3398 | 358.5484 | 4.59596 | 4.498705 | 5.322833 | 29 |
| 49 | M208T5_2 | 208.12 | 208.0613 | 208.1468 | 5.292748 | 5.122618 | 5.626582 | 20 |
| 50 | M367T6 | 367.3733 | 367.3262 | 367.4326 | 6.202497 | 5.306638 | 7.222815 | 17 |
| 51 | M312T5 | 312.2439 | 312.2324 | 312.2632 | 4.608397 | 4.553722 | 4.636218 | 9 |
| 52 | M226T7 | 226.2322 | 226.1679 | 226.2522 | 6.710986 | 6.216225 | 7.424545 | 20 |
| 53 | M426T8 | 426.4303 | 426.3808 | 426.4442 | 8.391491 | 8.077573 | 8.76924 | 10 |
| 54 | M172T5 | 172.162 | 172.0851 | 172.2384 | 4.737507 | 4.563005 | 5.034417 | 24 |
| 55 | M425T8 | 425.3743 | 425.3617 | 425.4302 | 8.385889 | 8.067898 | 8.771577 | 12 |
| 56 | M74T4_1 | 73.90972 | 73.86966 | 73.93328 | 4.090846 | 4.00084 | 4.132953 | 8 |
| 57 | M207T5 | 206.9649 | 206.9346 | 207.1138 | 4.710325 | 3.922137 | 5.026373 | 29 |
| 58 | M167T5 | 167.1145 | 167.0687 | 167.145 | 4.741767 | 4.582013 | 5.051045 | 29 |
| 59 | M135T5 | 135.071 | 135.0466 | 135.1469 | 4.844528 | 3.994998 | 5.335582 | 43 |

| | | | | | | | | |
|---|---|---|---|---|---|---|---|---|
| 60 | M146T5 | 146.0511 | 145.9879 | 146.1257 | 4.736136 | 3.879912 | 5.03403 | 24 |
| 61 | M280T7 | 280.1657 | 280.0732 | 280.2176 | 6.609518 | 6.107202 | 7.337065 | 22 |
| 62 | M187T6 | 187.1216 | 187.0467 | 187.1594 | 6.414293 | 5.835453 | 7.12864 | 23 |
| 63 | M200T5 | 200.1455 | 200.0629 | 200.1948 | 4.8877 | 4.591883 | 5.523827 | 41 |
| 64 | M179T5 | 179.0654 | 179.0532 | 179.1166 | 4.607565 | 4.487053 | 4.928165 | 18 |
| 65 | M92T4 | 91.97455 | 91.94838 | 92.03751 | 4.053925 | 3.982608 | 4.13534 | 7 |
| 66 | M278T6 | 278.3344 | 278.2616 | 278.3608 | 5.720584 | 5.433803 | 6.74317 | 18 |
| 67 | M406T7 | 406.4423 | 406.3948 | 406.4724 | 7.041523 | 6.689432 | 7.225967 | 10 |
| 68 | M188T5 | 188.1196 | 188.0529 | 188.158 | 4.677503 | 4.6082 | 5.007232 | 27 |
| 69 | M208T5_1 | 208.0663 | 208.0463 | 208.1471 | 4.638455 | 3.958748 | 4.957938 | 24 |
| 70 | M391T7 | 391.369 | 391.2347 | 391.4207 | 6.561392 | 6.304207 | 7.243367 | 14 |
| 71 | M177T5 | 177.0648 | 177.0473 | 177.1254 | 4.733226 | 4.308623 | 5.051045 | 30 |
| 72 | M220T5 | 220.1617 | 220.1399 | 220.2293 | 4.833999 | 4.57659 | 5.848925 | 32 |
| 73 | M317T5 | 317.3443 | 317.2859 | 317.4146 | 4.813825 | 4.6082 | 5.074 | 29 |
| 74 | M193T5 | 193.1525 | 193.1299 | 193.2315 | 4.759687 | 4.530382 | 5.093258 | 20 |
| 75 | M221T5 | 221.174 | 221.1657 | 221.2199 | 4.979858 | 4.926942 | 5.101042 | 9 |
| 76 | M316T5 | 316.3425 | 316.2198 | 316.3735 | 4.765487 | 4.584443 | 5.074 | 31 |
| 77 | M266T7 | 266.0434 | 266.0318 | 266.1316 | 6.691898 | 6.207917 | 7.440292 | 19 |
| 78 | M485T8 | 484.6534 | 484.6263 | 484.66 | 8.256312 | 8.025665 | 8.388897 | 8 |
| 79 | M255T7_2 | 255.2686 | 255.2362 | 255.3363 | 6.569836 | 6.115373 | 6.803218 | 20 |
| 80 | M225T6 | 225.2245 | 225.1354 | 225.268 | 6.071043 | 5.39591 | 7.424545 | 51 |
| 81 | M182T5 | 182.0719 | 182.0434 | 182.1318 | 4.567497 | 3.464748 | 4.947872 | 20 |
| 82 | M178T5 | 178.0756 | 177.9525 | 178.1363 | 4.755924 | 4.487053 | 5.084477 | 30 |
| 83 | M397T8 | 396.5288 | 396.4841 | 396.5543 | 8.246338 | 8.071815 | 8.421867 | 7 |
| 84 | M131T5 | 131.122 | 131.0508 | 131.1511 | 4.663193 | 4.4082 | 5.165063 | 17 |
| 85 | M72T6 | 72.05058 | 71.95451 | 72.16005 | 6.124783 | 5.333265 | 6.813032 | 21 |
| 86 | M263T5 | 263.2423 | 263.1701 | 263.2677 | 4.79075 | 4.613335 | 4.999563 | 16 |
| 87 | M176T5 | 176.049 | 175.9523 | 176.1152 | 4.919953 | 4.549835 | 6.001552 | 49 |
| 88 | M266T6 | 266.1447 | 266.0627 | 266.2094 | 5.510418 | 5.038627 | 5.659843 | 11 |
| 89 | M353T5 | 353.2452 | 353.2239 | 353.3185 | 5.140983 | 4.888225 | 5.492832 | 32 |
| 90 | M371T5 | 371.4163 | 371.3743 | 371.4595 | 5.171439 | 4.827015 | 5.389102 | 10 |

| | | | | | | | | |
|---|---|---|---|---|---|---|---|---|
| 91 | M369T7 | 369.3467 | 369.2537 | 369.3651 | 6.51023 | 6.08099 | 7.260225 | 17 |
| 92 | M106T4 | 105.9382 | 105.8733 | 106.0417 | 4.063212 | 3.52749 | 4.550438 | 26 |
| 93 | M357T5 | 357.4367 | 357.3631 | 357.455 | 4.977848 | 4.634423 | 5.787962 | 24 |
| 94 | M521T5 | 520.5782 | 520.5648 | 520.6259 | 4.854988 | 4.800562 | 5.19786 | 9 |
| 95 | M414T7 | 414.4479 | 414.335 | 414.4726 | 6.571032 | 6.041307 | 7.252895 | 14 |
| 96 | M172T6 | 172.1432 | 172.1199 | 172.1579 | 6.133707 | 5.827775 | 6.821057 | 15 |
| 97 | M202T5 | 202.17 | 202.1482 | 202.1797 | 4.990255 | 4.911543 | 5.126615 | 6 |
| 98 | M158T7 | 158.1448 | 158.1343 | 158.1624 | 7.120552 | 6.685003 | 7.369298 | 7 |
| 99 | M264T6 | 264.32 | 264.2702 | 264.3416 | 5.870879 | 5.476708 | 6.73376 | 12 |
| 100 | M352T8 | 352.4778 | 352.4684 | 352.5288 | 8.259575 | 8.04997 | 8.424112 | 9 |
| 101 | M244T5 | 244.2344 | 244.1522 | 244.292 | 4.867492 | 4.59545 | 5.43146 | 36 |
| 102 | M74T4_2 | 74.0312 | 73.94011 | 74.16742 | 4.302002 | 3.774872 | 5.499508 | 34 |
| 103 | M232T5 | 232.2348 | 232.1676 | 232.2619 | 4.807025 | 4.59147 | 4.993465 | 17 |
| 104 | M123T5 | 123.0017 | 122.9495 | 123.052 | 5.020699 | 4.887887 | 5.104255 | 8 |
| 105 | M335T5 | 335.2552 | 335.2374 | 335.2967 | 5.170685 | 4.860125 | 5.420178 | 17 |
| 106 | M460T5 | 459.5425 | 459.5342 | 459.547 | 4.996394 | 4.842767 | 5.226058 | 6 |
| 107 | M283T6 | 283.2785 | 283.2498 | 283.3304 | 5.859193 | 5.479027 | 6.525898 | 13 |
| 108 | M392T7 | 392.371 | 392.3567 | 392.3791 | 6.517585 | 6.32531 | 7.243367 | 7 |
| 109 | M213T6 | 213.1743 | 213.1318 | 213.2174 | 6.320772 | 5.855443 | 7.039818 | 8 |
| 110 | M434T6 | 434.4426 | 434.3655 | 434.5135 | 6.145999 | 6.00387 | 6.528655 | 10 |
| 111 | M387T6 | 387.3666 | 387.3378 | 387.4307 | 6.145427 | 5.941407 | 6.577873 | 11 |
| 112 | M449T7 | 449.4712 | 449.3931 | 449.5229 | 6.732345 | 6.222043 | 7.422218 | 19 |
| 113 | M365T5 | 365.2912 | 365.2337 | 365.3594 | 5.227072 | 4.63994 | 5.859082 | 25 |
| 114 | M376T6 | 376.3663 | 376.2666 | 376.387 | 5.655085 | 5.474603 | 6.717672 | 14 |
| 115 | M161T5 | 161.065 | 161.0378 | 161.1412 | 4.663825 | 4.614862 | 4.930335 | 20 |
| 116 | M134T5 | 134.1382 | 134.0785 | 134.1754 | 4.573527 | 4.462005 | 4.623803 | 12 |
| 117 | M324T6 | 324.2499 | 324.2319 | 324.315 | 5.674939 | 5.622702 | 6.153257 | 12 |
| 118 | M138T5 | 138.0558 | 137.9487 | 138.1698 | 4.501787 | 4.363835 | 4.524893 | 6 |
| 119 | M436T6 | 436.3484 | 436.254 | 436.4198 | 6.460165 | 5.870322 | 7.130917 | 21 |
| 120 | M432T6 | 432.3698 | 432.3265 | 432.5332 | 5.986876 | 4.724375 | 7.762662 | 74 |
| 121 | M329T8 | 329.2668 | 329.2553 | 329.3252 | 8.415417 | 8.089535 | 8.531017 | 8 |

| | | | | | | | | |
|---|---|---|---|---|---|---|---|---|
| 122 | M369T5 | 369.3536 | 369.3395 | 369.4205 | 5.393728 | 5.18054 | 5.521213 | 12 |
| 123 | M129T5 | 129.0513 | 128.9414 | 129.1696 | 4.631085 | 4.41297 | 5.365183 | 27 |
| 124 | M474T6 | 474.3243 | 474.2454 | 474.3546 | 6.172282 | 6.0024 | 6.239987 | 7 |
| 125 | M152T5 | 152.1082 | 152.0513 | 152.1497 | 4.88552 | 4.553662 | 5.036688 | 13 |
| 126 | M325T7 | 325.2715 | 325.2497 | 325.356 | 6.932663 | 6.523583 | 7.124052 | 6 |
| 127 | M326T5 | 326.3631 | 326.3312 | 326.4391 | 4.97847 | 4.653417 | 5.36114 | 27 |
| 128 | M126T6 | 125.9425 | 125.9265 | 125.9547 | 5.744497 | 5.24264 | 5.927697 | 17 |
| 129 | M260T9 | 260.2368 | 260.2297 | 260.253 | 8.885537 | 8.485632 | 9.349463 | 9 |
| 130 | M425T10 | 425.4083 | 425.3508 | 425.472 | 9.710659 | 8.891108 | 11.34222 | 40 |
| 131 | M229T5 | 229.221 | 229.1537 | 229.2941 | 4.840658 | 3.966078 | 5.249317 | 35 |
| 132 | M188T6 | 188.1492 | 188.1273 | 188.1645 | 6.295739 | 5.476708 | 6.914247 | 12 |
| 133 | M251T5 | 251.2539 | 251.2334 | 251.3257 | 4.865088 | 4.523812 | 5.290598 | 21 |
| 134 | M97T4 | 96.94113 | 96.88057 | 97.04735 | 3.980068 | 3.907642 | 4.189938 | 15 |
| 135 | M135T6 | 135.0588 | 135.0454 | 135.1108 | 5.78221 | 5.435147 | 6.724618 | 19 |
| 136 | M403T10 | 403.3655 | 403.3303 | 403.4249 | 9.630463 | 8.778365 | 10.50355 | 32 |
| 137 | M451T7 | 450.544 | 450.487 | 450.5634 | 6.697973 | 6.122972 | 7.196178 | 17 |
| 138 | M261T5 | 261.2362 | 261.1741 | 261.2645 | 4.654195 | 4.536012 | 5.039612 | 19 |
| 139 | M443T6 | 442.544 | 442.4419 | 442.6069 | 5.536385 | 4.964287 | 6.675498 | 43 |
| 140 | M386T6 | 386.4164 | 386.352 | 386.4801 | 6.468517 | 5.929533 | 7.269918 | 20 |
| 141 | M171T5 | 171.086 | 170.9592 | 171.1742 | 5.277487 | 4.015132 | 7.362828 | 84 |
| 142 | M265T5 | 265.2666 | 265.2534 | 265.3167 | 4.837291 | 4.636192 | 5.098895 | 16 |
| 143 | M749T5 | 748.7727 | 748.7487 | 748.8327 | 5.254344 | 5.204077 | 5.350302 | 6 |
| 144 | M433T6 | 433.3886 | 433.3475 | 433.5404 | 6.032262 | 4.724375 | 7.7479 | 52 |
| 145 | M426T10 | 426.4281 | 426.3636 | 426.4612 | 9.75558 | 8.871817 | 11.34222 | 33 |
| 146 | M435T6 | 435.3417 | 435.3231 | 435.3588 | 6.453475 | 6.215662 | 7.12864 | 15 |
| 147 | M303T6 | 303.3614 | 303.347 | 303.4293 | 5.787962 | 5.44812 | 6.721435 | 13 |
| 148 | M359T6 | 358.5296 | 358.4465 | 358.5658 | 6.128988 | 5.631097 | 7.187682 | 30 |
| 149 | M88T6 | 87.94592 | 87.90848 | 87.97305 | 6.428488 | 6.192818 | 7.1216 | 14 |
| 150 | M476T5 | 475.5246 | 475.4604 | 475.573 | 4.901722 | 4.723618 | 5.247163 | 15 |
| 151 | M416T7 | 416.3863 | 416.3646 | 416.4525 | 6.858348 | 6.534482 | 7.754625 | 15 |
| 152 | M147T5 | 147.0459 | 146.9527 | 147.1119 | 4.698943 | 4.48582 | 4.938027 | 27 |

| | | | | | | | | |
|---|---|---|---|---|---|---|---|---|
| 153 | M247T5 | 247.262 | 247.1802 | 247.297 | 4.948613 | 4.622613 | 5.816215 | 12 |
| 154 | M124T5_2 | 124.0537 | 124.026 | 124.0745 | 4.617241 | 4.528235 | 4.910857 | 12 |
| 155 | M335T6 | 335.2448 | 335.1874 | 335.4188 | 6.325805 | 5.920495 | 6.74287 | 14 |
| 156 | M257T5 | 257.2631 | 257.2351 | 257.3283 | 5.491283 | 4.604822 | 7.534892 | 90 |
| 157 | M431T5 | 431.4501 | 431.3795 | 431.529 | 4.697648 | 4.570842 | 5.134262 | 17 |
| 158 | M191T5 | 191.1229 | 191.0613 | 191.1579 | 4.798454 | 3.978033 | 5.848925 | 24 |
| 159 | M190T5 | 190.1178 | 189.9575 | 190.1404 | 4.865817 | 4.316508 | 5.03403 | 20 |
| 160 | M246T5 | 246.2288 | 246.0699 | 246.2807 | 4.87291 | 4.584443 | 6.030728 | 41 |
| 161 | M183T5 | 183.0446 | 182.9704 | 183.107 | 5.227072 | 4.616197 | 6.695352 | 65 |
| 162 | M156T5 | 156.0667 | 156.0481 | 156.1227 | 4.83406 | 4.622755 | 5.19075 | 19 |
| 163 | M450T6 | 449.5445 | 449.5118 | 449.5614 | 5.527583 | 5.377458 | 5.649298 | 7 |
| 164 | M189T5 | 189.1187 | 189.0748 | 189.1515 | 4.797717 | 4.524647 | 4.950863 | 11 |
| 165 | M163T5 | 163.1486 | 163.1231 | 163.1592 | 4.998885 | 4.553722 | 5.462115 | 13 |
| 166 | M196T5 | 196.0309 | 195.939 | 196.0693 | 5.14004 | 4.626225 | 5.875975 | 45 |
| 167 | M354T7 | 354.4442 | 354.3667 | 354.4621 | 6.746231 | 6.361625 | 8.034702 | 32 |
| 168 | M227T5 | 227.1832 | 227.1194 | 227.2382 | 5.018687 | 4.536012 | 6.267598 | 63 |
| 169 | M336T6 | 336.2591 | 336.2319 | 336.3717 | 6.400085 | 5.939828 | 6.759375 | 13 |
| 170 | M416T6 | 416.4134 | 416.3559 | 416.5353 | 5.908087 | 4.704952 | 6.422442 | 31 |
| 171 | M226T6 | 226.239 | 226.1662 | 226.2545 | 5.513625 | 4.91999 | 5.745368 | 12 |
| 172 | M298T5 | 298.3064 | 298.2472 | 298.3295 | 4.872331 | 4.829462 | 4.95576 | 6 |
| 173 | M240T7 | 240.2505 | 240.2396 | 240.2574 | 7.433231 | 6.949325 | 8.014493 | 8 |
| 174 | M212T5 | 212.1564 | 212.0792 | 212.1993 | 4.81497 | 4.597067 | 5.06108 | 15 |
| 175 | M195T5 | 195.1478 | 195.089 | 195.2011 | 4.820496 | 4.496517 | 5.076653 | 22 |
| 176 | M151T5 | 151.0715 | 151.0217 | 151.1328 | 4.618872 | 4.563142 | 4.922527 | 13 |
| 177 | M255T7_1 | 255.2946 | 255.227 | 255.3441 | 7.372452 | 6.828473 | 8.515738 | 47 |
| 178 | M290T6 | 290.332 | 290.2582 | 290.3571 | 5.629055 | 5.320175 | 6.215797 | 17 |
| 179 | M175T5 | 175.1521 | 175.0599 | 175.2679 | 4.582457 | 4.545042 | 4.744037 | 13 |
| 180 | M118T4 | 118.0675 | 118.0337 | 118.1517 | 4.460391 | 3.975738 | 4.576777 | 12 |
| 181 | M329T6 | 329.3526 | 329.2387 | 329.4026 | 5.651147 | 5.166517 | 6.499023 | 13 |
| 182 | M452T7 | 451.5617 | 451.5021 | 451.6147 | 6.74734 | 6.475758 | 7.432922 | 9 |
| 183 | M170T5 | 170.055 | 170.0454 | 170.1646 | 5.494567 | 5.312513 | 6.251442 | 9 |

| | | | | | | | | |
|---|---|---|---|---|---|---|---|---|
| 184 | M389T5 | 389.4323 | 389.3688 | 389.5051 | 5.119243 | 4.711592 | 5.64562 | 18 |
| 185 | M194T5 | 194.1305 | 194.0773 | 194.1728 | 4.879769 | 3.985343 | 5.816215 | 46 |
| 186 | M283T5 | 283.2747 | 283.2573 | 283.3279 | 4.855122 | 4.665015 | 5.206798 | 9 |
| 187 | M573T8 | 572.7379 | 572.6327 | 572.7564 | 8.047988 | 7.765975 | 8.287967 | 7 |
| 188 | M355T8 | 355.2292 | 355.1718 | 355.269 | 8.383578 | 8.217482 | 8.74599 | 18 |
| 189 | M265T6 | 265.2619 | 265.2378 | 265.2919 | 5.824332 | 5.48591 | 6.421703 | 13 |
| 190 | M420T8 | 420.3601 | 420.3222 | 420.4266 | 8.404088 | 8.077573 | 8.547208 | 11 |
| 191 | M415T6 | 415.3757 | 415.3375 | 415.5386 | 6.029583 | 4.716932 | 7.754625 | 69 |
| 192 | M210T6 | 210.2465 | 210.237 | 210.2644 | 5.602781 | 4.935188 | 5.966517 | 8 |
| 193 | M460T6 | 460.4621 | 460.3478 | 460.5523 | 6.008383 | 4.687955 | 7.102285 | 53 |
| 194 | M214T5 | 214.0388 | 214.0067 | 214.1741 | 4.736208 | 4.494853 | 4.922248 | 12 |
| 195 | M187T5 | 187.1374 | 187.0812 | 187.196 | 4.882978 | 4.487053 | 5.575695 | 48 |
| 196 | M209T5 | 209.188 | 209.098 | 209.304 | 4.998899 | 3.922137 | 6.897212 | 64 |
| 197 | M513T5 | 512.7247 | 512.6563 | 512.7331 | 5.253167 | 5.199528 | 5.78439 | 9 |
| 198 | M144T5 | 144.0477 | 143.9489 | 144.1152 | 4.59545 | 4.479872 | 5.097193 | 25 |
| 199 | M461T6 | 461.4652 | 461.35 | 461.5522 | 6.035273 | 5.462098 | 7.102285 | 25 |
| 200 | M404T10_2 | 404.4417 | 404.4364 | 404.4485 | 10.0536 | 9.561152 | 10.55363 | 10 |
| 201 | M160T5 | 160.0448 | 160.031 | 160.1436 | 4.897813 | 3.994998 | 5.416375 | 16 |
| 202 | M475T6 | 475.4706 | 475.362 | 475.5465 | 5.723953 | 5.426943 | 6.281918 | 11 |
| 203 | M306T6 | 306.3803 | 306.2188 | 306.4324 | 5.626352 | 4.981788 | 5.966517 | 7 |
| 204 | M385T9 | 385.3663 | 385.3549 | 385.4282 | 8.808473 | 8.30787 | 9.355553 | 11 |
| 205 | M284T5 | 284.3389 | 284.2731 | 284.3662 | 4.902873 | 4.626133 | 5.826607 | 19 |
| 206 | M372T5 | 372.384 | 372.3624 | 372.437 | 4.609092 | 4.571563 | 4.831875 | 14 |
| 207 | M215T5 | 215.1748 | 215.1396 | 215.2688 | 4.842089 | 4.298997 | 5.051045 | 10 |
| 208 | M328T5 | 328.3826 | 328.312 | 328.4441 | 4.822493 | 4.636192 | 5.848925 | 12 |
| 209 | M379T8 | 379.3869 | 379.3447 | 379.4502 | 8.220313 | 7.565443 | 9.349463 | 41 |
| 210 | M404T10_1 | 404.3776 | 404.312 | 404.4338 | 9.57431 | 8.780678 | 10.77525 | 28 |
| 211 | M264T5 | 264.2671 | 264.2537 | 264.274 | 4.720235 | 4.607517 | 4.96505 | 7 |
| 212 | M378T8 | 378.353 | 378.2687 | 378.4206 | 8.13723 | 7.379988 | 9.03398 | 43 |
| 213 | M503T7 | 503.4308 | 503.3626 | 503.4839 | 6.638138 | 6.178658 | 7.316297 | 18 |
| 214 | M114T5 | 114.031 | 113.9646 | 114.1785 | 5.046988 | 4.420903 | 6.55469 | 61 |

| | | | | | | | | |
|---|---|---|---|---|---|---|---|---|
| 215 | M362T8 | 362.3892 | 362.3476 | 362.4384 | 8.231772 | 7.781733 | 9.35787 | 28 |
| 216 | M353T6 | 353.2644 | 353.1886 | 353.3731 | 6.393686 | 6.054 | 7.258687 | 12 |
| 217 | M196T6 | 195.9413 | 195.887 | 196.0527 | 6.389887 | 5.979288 | 7.20758 | 12 |
| 218 | M384T5 | 384.475 | 384.377 | 384.5555 | 5.478747 | 5.17621 | 5.597997 | 11 |
| 219 | M156T6 | 156.1225 | 156.1128 | 156.1296 | 5.873973 | 5.571797 | 5.99175 | 6 |
| 220 | M111T6 | 111.0549 | 111.0427 | 111.122 | 5.742358 | 5.476708 | 6.267598 | 10 |
| 221 | M271T5 | 271.2721 | 271.2128 | 271.3206 | 5.01235 | 4.63171 | 5.656568 | 20 |
| 222 | M308T5 | 308.2241 | 308.1857 | 308.2746 | 4.73855 | 4.473382 | 4.755047 | 7 |
| 223 | M273T6 | 273.2275 | 273.121 | 273.3001 | 5.888452 | 5.57893 | 6.221482 | 11 |
| 224 | M224T5 | 224.022 | 223.8501 | 224.0541 | 5.154075 | 4.565117 | 6.679877 | 55 |
| 225 | M105T25 | 104.9667 | 104.8762 | 105.0455 | 24.63055 | 23.9696 | 24.93992 | 15 |
| 226 | M228T5 | 228.221 | 228.1191 | 228.2914 | 5.041906 | 4.512252 | 7.234532 | 68 |
| 227 | M399T8 | 399.3723 | 399.3594 | 399.4397 | 7.94449 | 7.76532 | 8.270852 | 10 |
| 228 | M355T7 | 355.4677 | 355.4437 | 355.5117 | 6.785228 | 6.675187 | 7.028888 | 9 |
| 229 | M354T6 | 354.2663 | 354.256 | 354.2781 | 6.300807 | 6.062613 | 6.940233 | 7 |
| 230 | M280T6 | 280.2175 | 280.1614 | 280.2848 | 5.610857 | 5.07418 | 6.015855 | 36 |
| 231 | M259T6 | 259.247 | 259.2185 | 259.2655 | 5.660023 | 5.524598 | 6.038915 | 13 |
| 232 | M127T6 | 126.9988 | 126.9693 | 127.0289 | 6.456532 | 6.020247 | 7.19737 | 9 |
| 233 | M387T7 | 387.3564 | 387.3427 | 387.3744 | 6.972148 | 6.799232 | 7.797917 | 8 |
| 234 | M565T5 | 564.6398 | 564.5613 | 564.6608 | 5.162512 | 4.815985 | 5.280285 | 9 |
| 235 | M332T6 | 332.438 | 332.353 | 332.5483 | 5.55463 | 4.760728 | 6.370675 | 25 |
| 236 | M259T10 | 259.234 | 259.172 | 259.2532 | 9.868698 | 9.35787 | 10.45108 | 19 |
| 237 | M435T5 | 435.3585 | 435.3199 | 435.4422 | 5.44581 | 5.04333 | 6.084603 | 33 |
| 238 | M105T5 | 104.9733 | 104.9417 | 105.0609 | 4.79096 | 4.548325 | 5.293372 | 15 |
| 239 | M199T5 | 199.1487 | 199.0631 | 199.2472 | 4.863142 | 4.604983 | 5.247163 | 15 |
| 240 | M140T5 | 139.9433 | 139.897 | 139.9651 | 5.291086 | 4.650345 | 7.20758 | 64 |
| 241 | M103T39 | 102.9748 | 102.8854 | 103.0464 | 38.84832 | 38.35487 | 39.58609 | 13 |
| 242 | M383T8 | 383.3468 | 383.2778 | 383.4302 | 8.143432 | 7.379988 | 9.35787 | 41 |
| 243 | M102T5 | 101.9594 | 101.9356 | 102.0502 | 4.615892 | 3.929412 | 4.910857 | 14 |
| 244 | M279T7_2 | 279.1445 | 279.1126 | 279.3053 | 6.517197 | 6.3692 | 6.780035 | 11 |
| 245 | M124T5_1 | 124.0433 | 124.0338 | 124.0528 | 5.475711 | 5.287843 | 6.017035 | 6 |

| | | | | | | | | |
|---|---|---|---|---|---|---|---|---|
| 246 | M100T5 | 100.0637 | 99.96779 | 100.1629 | 4.949728 | 4.404047 | 5.198827 | 10 |
| 247 | M155T5 | 155.0329 | 154.9533 | 155.1133 | 4.945187 | 4.109242 | 6.257265 | 60 |
| 248 | M219T6 | 219.2452 | 219.2335 | 219.2743 | 6.259818 | 5.848925 | 6.689968 | 17 |
| 249 | M272T5 | 272.2957 | 272.2031 | 272.3859 | 5.000985 | 4.41297 | 6.769828 | 65 |
| 250 | M330T5 | 330.4589 | 330.3412 | 330.5579 | 5.276552 | 4.610405 | 6.738723 | 61 |
| 251 | M158T5 | 158.1258 | 158.0591 | 158.2498 | 4.61727 | 3.814522 | 5.039612 | 22 |
| 252 | M281T6 | 281.2461 | 281.2213 | 281.284 | 6.016673 | 5.456118 | 6.636765 | 20 |
| 253 | M665T9 | 664.6787 | 664.6647 | 664.753 | 8.671957 | 8.196032 | 9.263683 | 13 |
| 254 | M437T6 | 437.3727 | 437.337 | 437.491 | 6.127657 | 5.162562 | 7.754625 | 57 |
| 255 | M384T8 | 384.3849 | 384.3452 | 384.4479 | 8.141454 | 7.565443 | 9.18486 | 36 |
| 256 | M361T8 | 361.3551 | 361.2737 | 361.4278 | 8.165892 | 7.55931 | 9.237092 | 41 |
| 257 | M365T6 | 365.2426 | 365.2244 | 365.2775 | 6.25881 | 5.995317 | 7.005225 | 9 |
| 258 | M256T6 | 256.2565 | 256.2409 | 256.3412 | 5.969001 | 5.667265 | 6.753462 | 12 |
| 259 | M420T10 | 420.3508 | 420.2641 | 420.426 | 9.745262 | 8.871817 | 11.33042 | 35 |
| 260 | M380T8 | 380.377 | 380.3492 | 380.4697 | 8.137378 | 7.557453 | 9.34716 | 17 |
| 261 | M577T9 | 576.6428 | 576.5682 | 576.7236 | 8.584213 | 8.198282 | 9.137933 | 16 |
| 262 | M744T8 | 743.5694 | 743.4783 | 743.65 | 7.935583 | 7.55931 | 8.485632 | 22 |
| 263 | M271T6 | 271.1649 | 271.0728 | 271.1802 | 6.000832 | 5.293717 | 6.60859 | 14 |
| 264 | M259T8 | 259.2351 | 259.1521 | 259.2582 | 7.930364 | 7.379988 | 8.544132 | 38 |
| 265 | M533T9 | 532.571 | 532.5509 | 532.6481 | 8.693738 | 8.206562 | 9.149787 | 12 |
| 266 | M181T5 | 180.9381 | 180.8342 | 181.0528 | 5.216848 | 4.121085 | 7.012478 | 62 |
| 267 | M185T8 | 185.0788 | 185.0436 | 185.1361 | 8.137378 | 7.55931 | 9.208072 | 37 |
| 268 | M146T6 | 146.0686 | 145.9677 | 146.1044 | 5.551391 | 5.320175 | 6.355525 | 12 |
| 269 | M88T5_2 | 87.99527 | 87.95979 | 88.063 | 5.489118 | 5.318035 | 5.601543 | 8 |
| 270 | M273T7 | 273.179 | 273.1183 | 273.307 | 6.641673 | 6.292673 | 7.490648 | 14 |
| 271 | M119T5 | 118.969 | 118.9469 | 119.0419 | 4.836427 | 4.013382 | 5.561873 | 11 |
| 272 | M504T7 | 504.4521 | 504.4322 | 504.4624 | 6.779329 | 6.45922 | 7.063242 | 6 |
| 273 | M329T10 | 329.2594 | 329.1856 | 329.2782 | 9.68801 | 8.883788 | 11.34222 | 27 |
| 274 | M278T5 | 278.2761 | 278.211 | 278.4009 | 4.808074 | 4.620492 | 5.340865 | 28 |
| 275 | M383T7 | 383.3439 | 383.2842 | 383.3539 | 7.051996 | 6.632302 | 7.211247 | 12 |
| 276 | M709T9 | 708.7437 | 708.673 | 708.7995 | 8.643965 | 8.195282 | 9.24475 | 14 |

| | | | | | | | | |
|---|---|---|---|---|---|---|---|---|
| 277 | M279T6 | 279.1926 | 279.0923 | 279.2609 | 5.62136 | 4.762678 | 6.248753 | 48 |
| 278 | M448T6 | 447.5505 | 447.4427 | 447.6552 | 5.777576 | 5.408868 | 6.454222 | 8 |
| 279 | M375T5 | 374.5352 | 374.4553 | 374.5653 | 5.225307 | 4.624082 | 6.758938 | 38 |
| 280 | M243T5 | 243.2403 | 243.1851 | 243.3583 | 4.95588 | 4.571563 | 6.717672 | 61 |
| 281 | M621T9 | 620.6581 | 620.5957 | 620.7746 | 8.648293 | 8.198282 | 9.277915 | 13 |
| 282 | M105T26 | 104.9312 | 104.876 | 104.9769 | 25.51545 | 25.22015 | 26.02356 | 13 |
| 283 | M753T9 | 752.7597 | 752.7352 | 752.8523 | 8.662337 | 8.17681 | 9.233933 | 11 |
| 284 | M173T6 | 173.0707 | 172.9439 | 173.1295 | 5.51022 | 5.312513 | 5.886313 | 9 |
| 285 | M279T7_1 | 279.2346 | 279.0966 | 279.3036 | 7.152774 | 6.871842 | 7.518357 | 18 |
| 286 | M165T5 | 165.0503 | 164.9516 | 165.1328 | 4.74451 | 4.470387 | 5.306073 | 21 |
| 287 | M297T6 | 297.3413 | 297.2508 | 297.3643 | 5.606466 | 5.389577 | 5.93751 | 12 |
| 288 | M448T10 | 448.4616 | 448.3934 | 448.5434 | 10.07723 | 9.40822 | 10.7569 | 13 |
| 289 | M407T8 | 407.4525 | 407.3832 | 407.5258 | 7.993823 | 6.874367 | 9.35787 | 41 |
| 290 | M406T8 | 406.4522 | 406.3628 | 406.5378 | 8.128526 | 7.370527 | 9.365162 | 44 |
| 291 | M304T5 | 304.4022 | 304.3281 | 304.4565 | 5.150543 | 3.94781 | 6.483412 | 50 |
| 292 | M233T6 | 233.1717 | 233.1307 | 233.2431 | 5.655197 | 5.417558 | 5.979752 | 8 |
| 293 | M159T5 | 159.0735 | 158.9445 | 159.1317 | 4.527725 | 3.057935 | 4.9321 | 25 |
| 294 | M242T5 | 242.2263 | 242.1127 | 242.2703 | 4.959802 | 4.489308 | 6.030728 | 50 |
| 295 | M384T7 | 384.3632 | 384.324 | 384.4114 | 7.149315 | 6.632302 | 7.467075 | 15 |
| 296 | M143T5 | 142.9448 | 142.8177 | 143.0298 | 4.774122 | 4.47596 | 5.41015 | 31 |
| 297 | M501T7 | 500.568 | 500.4794 | 500.6402 | 6.527504 | 5.997102 | 7.047365 | 14 |
| 298 | M387T5 | 387.3671 | 387.3469 | 387.5116 | 5.281855 | 5.133592 | 5.703458 | 18 |
| 299 | M309T5 | 309.2598 | 309.195 | 309.3687 | 4.994132 | 4.514453 | 6.407647 | 51 |
| 300 | M150T6 | 150.0447 | 150.0072 | 150.0755 | 5.501943 | 5.364565 | 5.780388 | 10 |
| 301 | M241T5 | 241.2309 | 241.1364 | 241.2566 | 4.991993 | 4.543827 | 6.225378 | 61 |
| 302 | M249T5 | 249.2296 | 249.0976 | 249.3052 | 5.069749 | 4.618872 | 6.753462 | 60 |
| 303 | M279T8 | 279.2579 | 279.1397 | 279.2859 | 8.151089 | 7.6323 | 9.414298 | 30 |
| 304 | M269T6 | 269.2736 | 269.1457 | 269.2808 | 5.891531 | 5.763825 | 6.178635 | 6 |
| 305 | M87T5 | 87.04444 | 86.94773 | 87.11876 | 4.937164 | 4.38929 | 5.9054 | 30 |
| 306 | M166T6 | 166.0692 | 166.052 | 166.0835 | 5.578662 | 5.402183 | 5.946 | 10 |
| 307 | M259T7 | 259.223 | 259.1248 | 259.2573 | 7.006599 | 6.28361 | 7.22236 | 18 |

| | | | | | | | | |
|---|---|---|---|---|---|---|---|---|
| 308 | M363T6 | 363.4557 | 363.4366 | 363.5776 | 5.667163 | 5.271137 | 6.759375 | 16 |
| 309 | M385T7 | 385.3705 | 385.3585 | 385.4628 | 7.03265 | 6.625083 | 7.42183 | 11 |
| 310 | M274T5 | 274.3445 | 274.2299 | 274.4143 | 4.886673 | 3.694202 | 6.759375 | 70 |
| 311 | M269T5 | 269.257 | 269.1977 | 269.3185 | 4.837958 | 4.568637 | 5.470238 | 23 |
| 312 | M745T7 | 744.5654 | 744.4746 | 744.6621 | 7.069075 | 6.632302 | 7.382305 | 11 |
| 313 | M268T5 | 268.2406 | 268.1213 | 268.279 | 4.866782 | 4.534728 | 5.263162 | 27 |
| 314 | M90T4 | 89.98106 | 89.93311 | 90.05494 | 3.672078 | 3.452963 | 3.84648 | 10 |
| 315 | M744T7 | 743.5469 | 743.4645 | 743.6116 | 7.149315 | 6.632302 | 7.467075 | 15 |
| 316 | M136T5 | 136.0728 | 136.0132 | 136.1309 | 4.833405 | 4.528235 | 5.69791 | 35 |
| 317 | M391T5 | 391.3636 | 391.3539 | 391.448 | 5.466252 | 5.159352 | 5.680545 | 9 |
| 318 | M230T5 | 230.2741 | 230.2202 | 230.337 | 4.878348 | 3.708623 | 6.181288 | 64 |
| 319 | M70T5 | 70.26607 | 70.18392 | 70.37566 | 4.585253 | 3.637205 | 4.696098 | 30 |
| 320 | M168T5 | 168.0326 | 167.9553 | 168.1103 | 5.302423 | 4.648085 | 6.677562 | 65 |
| 321 | M346T5 | 346.4617 | 346.3345 | 346.5588 | 5.228157 | 4.548605 | 6.343247 | 42 |
| 322 | M310T5 | 310.2738 | 310.2294 | 310.3458 | 4.918743 | 4.630575 | 5.76445 | 21 |
| 323 | M300T5 | 300.3557 | 300.2833 | 300.4228 | 4.901567 | 4.536012 | 6.17901 | 50 |
| 324 | M149T5 | 149.0866 | 149.0561 | 149.1404 | 4.752138 | 4.426958 | 5.08235 | 25 |
| 325 | M261T3 | 261.1536 | 261.1129 | 261.2276 | 3.451061 | 3.381335 | 3.601138 | 16 |
| 326 | M355T5 | 355.2696 | 355.2425 | 355.3793 | 5.139967 | 4.888225 | 5.463988 | 27 |
| 327 | M245T5 | 245.2425 | 245.1004 | 245.258 | 4.887887 | 4.597067 | 5.373245 | 23 |
| 328 | M406T6 | 406.4855 | 406.4605 | 406.5404 | 5.582498 | 5.35197 | 6.122972 | 9 |
| 329 | M318T5 | 318.421 | 318.3172 | 318.503 | 4.879078 | 3.766963 | 6.142183 | 68 |
| 330 | M362T5 | 362.4546 | 362.3381 | 362.5408 | 4.8569 | 3.808715 | 6.220325 | 59 |
| 331 | M258T5 | 258.3242 | 258.2516 | 258.3995 | 4.943578 | 4.564758 | 7.032885 | 54 |
| 332 | M275T5 | 275.3404 | 275.2624 | 275.407 | 4.879197 | 3.895902 | 6.00717 | 53 |
| 333 | M319T5 | 319.4282 | 319.3143 | 319.459 | 4.861863 | 3.760945 | 6.144503 | 49 |
| 334 | M157T5 | 157.0629 | 156.972 | 157.1355 | 4.644663 | 3.791175 | 5.024163 | 32 |
| 335 | M340T5 | 340.4178 | 340.3283 | 340.4631 | 4.983783 | 4.05034 | 6.74317 | 61 |
| 336 | M180T5 | 180.1089 | 180.0466 | 180.1586 | 4.801418 | 4.504365 | 5.772545 | 34 |
| 337 | M286T5 | 286.3582 | 286.2198 | 286.4336 | 5.06582 | 4.600172 | 5.923632 | 36 |
| 338 | M116T4 | 116.028 | 115.9661 | 116.0476 | 4.451567 | 3.781553 | 4.645288 | 11 |

| | | | | | | | | |
|---|---|---|---|---|---|---|---|---|
| 339 | M477T3 | 477.3555 | 477.284 | 477.4262 | 3.45844 | 3.422433 | 4.00606 | 19 |
| 340 | M225T5 | 225.22 | 225.076 | 225.2736 | 4.967244 | 4.584443 | 5.385685 | 40 |
| 341 | M361T7 | 361.3473 | 361.2736 | 361.3599 | 7.154875 | 6.641883 | 7.476693 | 16 |
| 342 | M255T5 | 255.2469 | 255.2051 | 255.4091 | 5.330778 | 4.630575 | 6.017035 | 34 |
| 343 | M378T7 | 378.3466 | 378.2763 | 378.3965 | 7.07633 | 6.644183 | 7.237908 | 13 |
| 344 | M478T3 | 478.3972 | 478.3304 | 478.471 | 3.454278 | 3.421262 | 3.736215 | 20 |
| 345 | M362T7 | 362.3718 | 362.306 | 362.4329 | 7.056781 | 6.634605 | 7.235597 | 12 |
| 346 | M185T7 | 185.0724 | 185.0551 | 185.0954 | 7.159033 | 6.644183 | 7.474392 | 15 |
| 347 | M379T7 | 379.366 | 379.329 | 379.4061 | 7.156033 | 6.644183 | 7.484 | 16 |
| 348 | M184T5 | 184.0849 | 184.0628 | 184.1326 | 5.27846 | 4.635747 | 7.20758 | 64 |
| 349 | M239T3 | 239.1385 | 239.0578 | 239.1741 | 3.481778 | 3.433178 | 4.014758 | 35 |
| 350 | M354T5 | 354.2597 | 354.1945 | 354.4242 | 5.150358 | 4.829887 | 5.61681 | 36 |
| 351 | M559T6 | 558.6428 | 558.5846 | 558.6655 | 5.519207 | 5.115992 | 5.606415 | 9 |
| 352 | M501T5 | 500.5765 | 500.5425 | 500.6465 | 5.446273 | 4.95971 | 5.8206 | 23 |
| 353 | M256T5 | 256.2599 | 256.2145 | 256.3263 | 4.855108 | 4.613167 | 5.566637 | 37 |
| 354 | M302T5 | 302.4176 | 302.3474 | 302.4954 | 4.937109 | 4.528235 | 6.340997 | 58 |

**Supplementary Table 4.** Identification of metabolic pathways associated by Thymoquinone treatment in (A) HL-60 and (B) Jurkat.

**A. HL-60**

| Pathway Name | Hits | FDR | Impact |
|---|---|---|---|
| Linoleic acid metabolism | 8 | 1 | 0.88541 |
| Phenylalanine metabolism | 18 | 1 | 0.41824 |
| D-Arginine and D-ornithine metabolism | 4 | 1 | 0.5 |
| Arachidonic acid metabolism | 21 | 1 | 0.27959 |
| Arginine and proline metabolism | 24 | 1 | 0.51252 |
| Glycine, serine and threonine metabolism | 13 | 1 | 0.29809 |
| Valine, leucine and isoleucine biosynthesis | 7 | 1 | 0.26921 |
| Tyrosine metabolism | 20 | 1 | 0.24942 |
| beta-Alanine metabolism | 7 | 1 | 0.12579 |
| Sulfur metabolism | 4 | 1 | 0.26693 |
| Phenylalanine, tyrosine and tryptophan biosynthesis | 6 | 1 | 0.0818 |
| Pantothenate and CoA biosynthesis | 6 | 1 | 0.30985 |
| Biotin metabolism | 2 | 1 | 0.19512 |
| Caffeine metabolism | 4 | 1 | 0.18429 |
| Nitrogen metabolism | 8 | 1 | 0.00763 |
| Vitamin B6 metabolism | 6 | 1 | 0.31289 |
| Alanine, aspartate and glutamate metabolism | 4 | 1 | 0.05128 |
| Citrate cycle (TCA cycle) | 3 | 1 | 0.18952 |
| D-Glutamine and D-glutamate metabolism | 1 | 1 | 0 |
| Cysteine and methionine metabolism | 10 | 1 | 0.33174 |
| Primary bile acid biosynthesis | 8 | 1 | 0.2378 |
| Tryptophan metabolism | 15 | 1 | 0.33769 |
| Pentose and glucuronate interconversions | 9 | 1 | 0.31882 |
| Glycerophospholipid metabolism | 6 | 1 | 0.30632 |
| Thiamine metabolism | 3 | 1 | 0.12481 |

| | | | |
|---|---|---|---|
| Nicotinate and nicotinamide metabolism | 7 | 1 | 0.19348 |
| Ubiquinone and other terpenoid-quinone biosynthesis | 5 | 1 | 0.16161 |
| Taurine and hypotaurine metabolism | 2 | 1 | 0 |
| Glycosylphosphatidylinositol (GPI)-anchor biosynthesis | 1 | 1 | 0.0439 |
| Lysine biosynthesis | 4 | 1 | 0.10418 |
| Histidine metabolism | 6 | 1 | 0.2664 |
| Glyoxylate and dicarboxylate metabolism | 7 | 1 | 0.19124 |
| Valine, leucine and isoleucine degradation | 5 | 1 | 0.03378 |
| Cyanoamino acid metabolism | 1 | 1 | 0 |
| Galactose metabolism | 5 | 1 | 0.11505 |
| Lysine degradation | 6 | 1 | 0.17953 |
| Fructose and mannose metabolism | 6 | 1 | 0.13693 |
| Pyruvate metabolism | 3 | 1 | 0 |
| Pentose phosphate pathway | 3 | 1 | 0.04289 |
| Pyrimidine metabolism | 8 | 1 | 0.13121 |
| Ascorbate and aldarate metabolism | 5 | 1 | 0.13739 |
| Butanoate metabolism | 4 | 1 | 0.09111 |
| Propanoate metabolism | 3 | 1 | 0.00164 |
| Riboflavin metabolism | 1 | 1 | 0 |
| alpha-Linolenic acid metabolism | 2 | 1 | 0.20335 |
| Aminoacyl-tRNA biosynthesis | 10 | 1 | 0 |
| Ether lipid metabolism | 1 | 1 | 0.03523 |
| Glycolysis or Gluconeogenesis | 2 | 1 | 0 |
| Glutathione metabolism | 3 | 1 | 0.0019 |
| Glycerolipid metabolism | 2 | 1 | 0.00714 |
| Inositol phosphate metabolism | 3 | 1 | 0.01203 |
| Limonene and pinene degradation | 6 | 1 | 0.14323 |
| Fatty acid elongation in mitochondria | 1 | 1 | 0 |
| Fatty acid biosynthesis | 3 | 1 | 0 |
| Starch and sucrose metabolism | 3 | 1 | 0.01265 |

| | | | |
|---|---|---|---|
| Fatty acid metabolism | 3 | 1 | 0.04482 |
| Drug metabolism - other enzymes | 1 | 1 | 0.05863 |
| Folate biosynthesis | 1 | 1 | 0.03372 |
| Purine metabolism | 6 | 1 | 0.03726 |
| Steroid hormone biosynthesis | 7 | 1 | 0.04156 |
| Amino sugar and nucleotide sugar metabolism | 5 | 1 | 0.00272 |
| Metabolism of xenobiotics by cytochrome P450 | 1 | 1 | 0 |
| Drug metabolism - cytochrome P450 | 2 | 1 | 0.01613 |
| Porphyrin and chlorophyll metabolism | 4 | 1 | 0.021 |

**B. Jurkat**

| Pathway Name | Hits | FDR | Impact |
|---|---|---|---|
| Arachidonic acid metabolism | 14 | 0.1495 | 0.27078 |
| Tyrosine metabolism | 15 | 0.20894 | 0.14394 |
| Vitamin B6 metabolism | 5 | 1 | 0.23812 |
| Alanine, aspartate and glutamate metabolism | 3 | 1 | 0.08547 |
| Synthesis and degradation of ketone bodies | 1 | 1 | 0.7 |
| Taurine and hypotaurine metabolism | 2 | 1 | 0.07014 |
| Caffeine metabolism | 2 | 1 | 0 |
| Riboflavin metabolism | 2 | 1 | 0.10178 |
| Histidine metabolism | 4 | 1 | 0.19919 |
| Steroid hormone biosynthesis | 9 | 1 | 0.09715 |
| Propanoate metabolism | 3 | 1 | 0.09079 |
| Primary bile acid biosynthesis | 4 | 1 | 0.12731 |
| Glyoxylate and dicarboxylate metabolism | 4 | 1 | 0.17092 |
| Phenylalanine, tyrosine and tryptophan biosynthesis | 2 | 1 | 0.00062 |
| Valine, leucine and isoleucine biosynthesis | 2 | 1 | 0.11871 |
| Butanoate metabolism | 3 | 1 | 0.09938 |
| beta-Alanine metabolism | 2 | 1 | 0.01119 |
| Cysteine and methionine metabolism | 4 | 1 | 0.0673 |

| | | | |
|---|---|---|---|
| Fatty acid biosynthesis | 3 | 1 | 0 |
| Citrate cycle (TCA cycle) | 1 | 1 | 0.04361 |
| Fatty acid metabolism | 3 | 1 | 0.04482 |
| Ubiquinone and other terpenoid-quinone biosynthesis | 2 | 1 | 0.11172 |
| Nitrogen metabolism | 2 | 1 | 0 |
| Ether lipid metabolism | 1 | 1 | 0.03523 |
| Valine, leucine and isoleucine degradation | 2 | 1 | 0.03938 |
| Sphingolipid metabolism | 1 | 1 | 0.29423 |
| Fatty acid elongation in mitochondria | 1 | 1 | 0 |
| Pantothenate and CoA biosynthesis | 1 | 1 | 0.05124 |
| Lysine degradation | 2 | 1 | 0.03404 |
| Glycine, serine and threonine metabolism | 2 | 1 | 0.0037 |
| Tryptophan metabolism | 4 | 1 | 0.08393 |
| Pentose phosphate pathway | 1 | 1 | 0 |
| Pyruvate metabolism | 1 | 1 | 0 |
| Lysine biosynthesis | 1 | 1 | 0.0423 |
| Aminoacyl-tRNA biosynthesis | 3 | 1 | 0 |
| Glycerophospholipid metabolism | 1 | 1 | 0.04571 |
| Arginine and proline metabolism | 3 | 1 | 0.03701 |
| Nicotinate and nicotinamide metabolism | 1 | 1 | 0.10565 |
| Ascorbate and aldarate metabolism | 1 | 1 | 5.00E-04 |
| Phenylalanine metabolism | 1 | 1 | 0.11906 |
| Pyrimidine metabolism | 1 | 1 | 0 |
| Purine metabolism | 2 | 1 | 0.02202 |
| Porphyrin and chlorophyll metabolism | 1 | 1 | 0.00331 |
